# Defined Diets Link Iron and α-Linolenic Acid to Cyp1b1 Regulation of Neonatal Liver Development Through Srebp Forms and LncRNA H19

**DOI:** 10.3390/ijms26052011

**Published:** 2025-02-25

**Authors:** Colin R. Jefcoate, Michele C. Larsen, Yong-Seok Song, Meghan Maguire, Nader Sheibani

**Affiliations:** 1 Department of Cell and Regenerative Biology, University of Wisconsin School of Medicine and Public Health, Madison, WI 53705, USA; mlarsen@wisc.edu (M.C.L.); mlmaguire@wisc.edu (M.M.); 2 Department of Ophthalmology and Visual Sciences, University of Wisconsin School of Medicine and Public Health, Madison, WI 53705, USA; song224@wisc.edu

**Keywords:** miRNA, LncRNA, Vitamin A, iron homeostasis, sexual dimorphism, P450 cytochromes, liver development

## Abstract

*Cyp1b1* substantially affects hepatic vascular and stellate cells (HSC) with linkage to liver fibrosis. Despite minimal hepatocyte expression, *Cyp1b1* deletion substantially impacts liver gene expression at birth and weaning. The appreciable *Cyp1b1* expression in surrounding embryo mesenchyme, during early organogenesis, provides a likely source for *Cyp1b1*. Here defined breeder diets established major interconnected effects on neonatal liver of α-linolenic acid (ALA), vitamin A deficiency (VAD) and suboptimal iron fed mice. At birth *Cyp1b1 deletion* and VAD each activated perinatal HSC, while suppressing iron control by hepcidin. *Cyp1b1* deletion also advanced the expression of diverse genes linked to iron regulation. Postnatal stimulations of *Srebp*-regulated genes in the fatty acid and cholesterol biosynthesis pathways were suppressed by *Cyp1b1*-deficiency. *LncRNA H19* and the neutrophil alarmin *S100a9* expression increased due to slower postnatal decline with *Cyp1b1* deficiency. VAD reversed each of *Cyp1b1* effect, probably due to enhanced HSC release of Apo-Rbp4. At birth, *Cyp1b1* deletion enhanced *H19* participation. Notably, a suppressor (*Cnot3*) decreased while an activity partner (*Ezh2*/H3K methylation) increased *H19* expression. ALA elevated hepcidin mRNA and countered the inhibitory effects of *Cyp1b1* deletion on hepcidin expression. Oxylipin metabolites of ALA from highly expressed hepatic *Cyps* are potential mediators. *Cyp* expression patterns demonstrated female dimorphism for neonatal liver. Mothers followed one of three fetal growth support programs probably linked to maturity at conception.

## 1. Introduction

Cyp1b1 is a unique Cytochrome P450 that commonly modulates cells remotely through the effects of its metabolites and integration with inflammatory factors, including cytokines and microRNAs (miRNAs) [1,2,3,4]. Cyp1b1 is regulated by several 5′-promoter regulatory regions located far from the transcription start site, and by miRNAs at sites in a long, conserved 3′-UTR. Cyp1b1 metabolism provides local modulation of mesenchymal, vascular and hematopoietic lineages that respond to redox signaling and control the production of reactive oxygen species (ROS) [5,6]. Deletion of Cyp1b1 (*Cyp1b1^−/−^*) in these cells increases ROS. Local cell functions respond to oxidative modifications and to changes in the expression and organization of extracellular matrix (ECM) proteins. N-acetylcysteine’s reversal of these changes is an indicator of such regulation.

Cyp1b1 is highly expressed in mouse embryos mid-gestation [7,8]. Notably, *Cyp1b1* co-locates at several sites of retinoic acid developmental activity. Cyp1b1 functions with P450 oxidoreductase and NADPH to convert retinol to retinoic acid [7,8]. However, the dominant synthesis pathway utilizes NAD^+^ via *Radh10* and *Aldh1a2* [9]. Local oxygen and metabolic support are very different for Cyp1b1 and NADPH/O_2_ [10].

Constitutive Cyp1b1 activation occurs through multiple mechanisms. In C3H10T1/2 cells, β-catenin and AhR are released together from cadherin junctional complexes [11]. This stimulation matches the direct ligand activation of AhR [10,12]. Cyp1b1 substrates also elevate the protein by slowing the normally rapid turnover [13]. Specific miRNAs enhance mRNA turnover and block translation [14]. In addition, endothelial cells (ECs) are activated by shear flow or oxygen changes [6,15], and hepatic stellate cells (HSCs) merely by plating [5], while breast epithelia change their expression either during capillary tubule formation or mechanical surface stretching [16].

Human primary congenital glaucoma (PCG) results from over one hundred CYP1B1 coding mutations that disrupt ocular trabecular meshwork (TM) integrity and function [7,16,17,18]. In human eyes, CYP1B1 is well expressed in epithelial cells but has very little expression in the trabecular meshwork ECs [19]. We have used lineage-specific *Cyp1b1* deletions from cells present in the mouse eye. Cyp1b1 in vascular ECs, pericytes, and astrocytes each impacted structural features of the TM tissue that obstructs ocular fluid outflow that defines PCG [4,19].

Expression of *Cyp1b1* in human and mouse livers has a far greater association with HSCs than with hepatocytes. These cells repair tissue damage but also generate fibrosis in many disease states [5,20]. HSCs are also the dominant source of stored vitamin A/retinol. However, *Cyp1b1*-deletion and vitamin A deficiency (VAD) each activate HSC to increase synthesis and release of cell support extracellular matrix proteins (ECM). In culture, *Cyp1b1* expression in HSC increases extensively over several days [5,20]. This expression is also remarkably labile. *Cyp1b1* is also the dominant *Cyp* in bone marrow mesenchymal progenitor cells [1,2], in liver sinusoidal ECs [21], and in retinal pericytes [22]. *Cyp1b1* is expressed selectively in the cells of monocyte lineages, particularly interleukin (IL)-13-induced peritoneal macrophages [1]. For each cell source, selective functions have been established.

Mid-gestation, hepatoblast markers and primitive cell assemblies appear in the septum transverse mesenchyme (STM) that is adjacent to the heart [7,19]. The STM expresses appreciable *Cyp1b1* [8]. This primitive liver supports the heart by delivering erythropoiesis with a key role in iron homeostasis. The STM comprises cells that are closely related to pericytes and HSC [23,24,25,26]. The high levels of Cyp1b1 in the STM where the liver is expanding over the next 3 days is striking [8]. In the next 7 days leading up to birth, distinct liver lobules form, comprising plates of hexagonal hepatocytes interspersed by the portal triad of oxygen-rich hepatic artery, portal vein, and bile duct [23]. The oxygen gradient from low in the central vein, to high in the portal triad, is of key relevance to Cyp1b1 participation.

Our previous work suggested that Cyp1b1 expression may impact liver development through regulation of dietary iron [27]. The major iron regulator Hepcidin (HepC) is a peptide that is delivered from a larger pro-hepcidin by the *Hamp* gene [28]. HepC regulates iron by targeting the unique iron transporter ferroportin (Fpn) for degradation. Fpn increases circulating iron in two ways: in specialized macrophages, it exports iron that accumulates from degradation of erythrocytes, and in the gut epithelia it transports dietary iron. Erythropoiesis is an important part of iron homeostasis [29]. Hamp expression provides a measure of circulating iron levels. The *Hamp–Fpn* pairing also functions in EC as a vascular iron transport system, notably in the brain and retina [30]. In hepatocytes, *Fpn* polarizes associated macrophages (*Fpn*^+^/M1 and *Fpn*^−^/M2) [31].

At birth, *Cyp1b1*^−/−^ and VAD diet each suppress Hamp mRNA. HSC release retinol to sustain constant blood retinol levels, even as liver levels decline with VAD. HSCs transfer retinol into hepatocytes as retinal binding protein 4 (Rbp4) complexes with the Stra6 surface receptor [32]. Recent work showed that this transfer activates Srebp isoforms. A striking feature of the liver growth from birth to weaning is that genes regulated by Srebp isoforms are collectively highly responsive, including genes from pathways for fatty acid (*Srebp1*) and cholesterol (*Srebp2*) biosynthesis. Included in the *Srebp1* set are *Hamp1* and *Hamp2* [33]. The over thirty *Srebp* targeted genes that increased in WT mice were no longer increased in *Cyp1b1*^−/−^ liver.

To study the effect of retinol depletion, we replaced the optimized breeder diet (Tekland BD2019) with a defined LF12 diet. This diet lacked iron supplementation and used a fat source that lacked α-linolenic acid (ALA), the major ω3-unsaturated fatty acid in a soyabean-based BD diet. We examined LF12 with severe retinol depletion (LF12-VAD) and also tested the restoration of, respectively, iron or α-linolenic acid (ALA) to BD levels (LF12-Fe and LF12-ALA) [8,34]. The expression analyses at birth showed a very selective overlap of *Cyp1b1* deletion and VAD on HSC activation and suppression of hepcidin. For animals fed the LF12 diet, major neonatal increases in *Srebp*-regulation were completely dependent on *Cyp1b1* [8]. This regulation exhibits distinctive modulation by retinol that implicates HSCs. ALA is a natural substrate for Cyp1b1 [35], with metabolites functioning in diverse ways, including as peroxisome proliferator-activated receptor (PPAR) and Ca-channel activators.

*Cyp1b1* has little expression in mouse hepatocytes but functions extensively in ECs, pericytes, and various inflammatory cells of the monocyte lineage. Bone morphogenetic protein 6 (BMP6) is the key stimulant for *Hamp* [30,36]. This cytokine is delivered from liver sinusoidal ECs (LSECs) in response to the uptake of circulating iron. Much of the circulating iron is bound by transferrin, which is taken up by transferrin receptors (*Tfrc1*) into the hepatocyte endosome network. However, in ECs iron uptake is independent of *Tfr1* [36] but is stimulated by estrogen receptor α (ERα) and estradiol (E2), a Cyp1b1 substrate. In retinal ECs, *Cyp1b1* deficiency elevates E2 levels, ROS, and NF-κB signaling [1,4,37]. The ROS program, which includes major suppression of eNOS, and release of the ECM protein thrombospondin-2 depends on the oxygen concentration and is reversed by N-acetylcysteine. In the liver, iron uptake by SLECs not only activates NF-κB but also Nrf2, a direct stimulant of BMP6. High levels of multiple *Cyps* in hepatocytes can participate in ROS metabolism in the neonatal liver. The functions of *Cyp1b1* need to be considered in light of this overwhelming background of overlapping metabolism activity. We propose *Cyp1b1* functions to modify highly local levels of steroids and oxylipins.

*Cyp1b1* regulation may control neonatal gene expression through a set of three genes (*Afp*, insulin-like growth factor 2 (*Igf2*), and *H19*) that mark hepatoblasts and are each regulated by *Cnot*3, an initiator of polyA de-adenylation [38]. Each is near to its maximum before birth. *Afp* and *Igf*2 decline severely from postnatal day 3 (P3) to weaning (P21). H19 deletion slows down both neonatal and adult liver development [39]. Each is elevated at weaning in *Cyp1b1*^−/−^ mice. The LncRNA *H19* is suppressed by direct interaction with de-adenylation activator *Cnot3*. *H19* directly promotes H3K methylator *Evh2*. We show that each *H19* modifier is appropriately affected at birth, consistent with elevation of *H19* expression [39,40].

We have discovered that *H19*, *Afp*, and several other functionally important genes share not only neonatal stimulation in *Cyp1b1*^−/−^ cells but also reversals when this deletion is combined with VAD. This intervention is likely to arise from altered HSC activity. This integration extends to all *Srebp* suppressions. Previous research [2,41,42] has shown that *Cyp1b1* deletion suppresses adult obesity and redirects sexual bias in gene expression that is linked to growth hormone (GH) signaling [43]. Many individual forms of *Cyps*, particularly those regulated by lipid ligands (PPARα, CAR, PXR, and Hnf4α), exhibit distinctive dimorphism that has been linked to adult obesity. This characterization shows that the liver has female dimorphism prior to weaning. Here, we provide evidence that *Cyp1b1’s* effects on neonatal processes may influence these adult processes.

We use modest changes in defined breeder diets in comparison to optimized breeder diets to probe the effects of *Cyp1b1* deletion on the liver growth and gene expression. The precision of the *Cyp1b1* and dietary effects on neonates suggests that the primary target is the central metabolism regulator mTORC1, and that it is coupled with the Cyp1b1 metabolism. Cyp1b1 partners with retinol and iron in pre-pubertal development. Based on individual litters and littermates, it has been established that the nulliparous mothers exert clustered effects that are shared by each pup independent of the sex. The retinol/Cyp1b1 partnership that is identified here emphasizes functional integration of *Cyp1b1* and *H19* with direct complexes with *Cnot3* and *Evh2*, respectively. In complementary research we are examining how such pre-pubertal changes impact adult functions, notably metabolic homeostasis, nonalcoholic fatty liver disease (NAFLD) and obesity.

Synthesis of long chain fatty acids from Acetyl-CoA under the control of *Srebp1* is almost absent at birth [8]. This multigene process appears to be dependent on *Cyp1b1* during neonatal development. Dietary ALA is the principle dietary precursor of arachidonic acid (AA), which then generates numerous functionally active oxylipins that mostly comprise Prostaglandins, Leukotrienes, and Epoxyeicosatrienoic acids (EETs). Meta studies on the dietary impacts of ALA have recently been expertly reviewed [44,45]. EETs include ligands for PPARγ, TRPV1 and other receptors or channels [46]. Transience for these EETs’ effects is conferred in two ways. The turnover of *Cyp1b1* is rapid, caused by the specific miRNA that target the 3′-UTR and by high levels of local epoxide hydrolase.

Here, we present evidence that *Cyp1b1* deletion greatly advances clustered expression of over 100 genes that promote iron availability (*Trf*, *Ftl* forms), control of ferroptosis (*Gpx4*) and elevate AA synthesis (*Fasm1*). We also show that extreme retinol deficiency (VAD) produces scarcely any perinatal changes that are not linked to *Cyp1b1*. We attribute this dual regulation to HSCs. We contrast the specificity and transience of *Cyp1b1* in neonatal liver support cells (ECs, HSCs, and monocytes) with the highly expressed female dimorphic *Cyps* that generate a constant oxylipin-rich environment for liver development and growth [47].

## 2. Results

### 2.1. Project Rationale and Design

**Figure 1:**
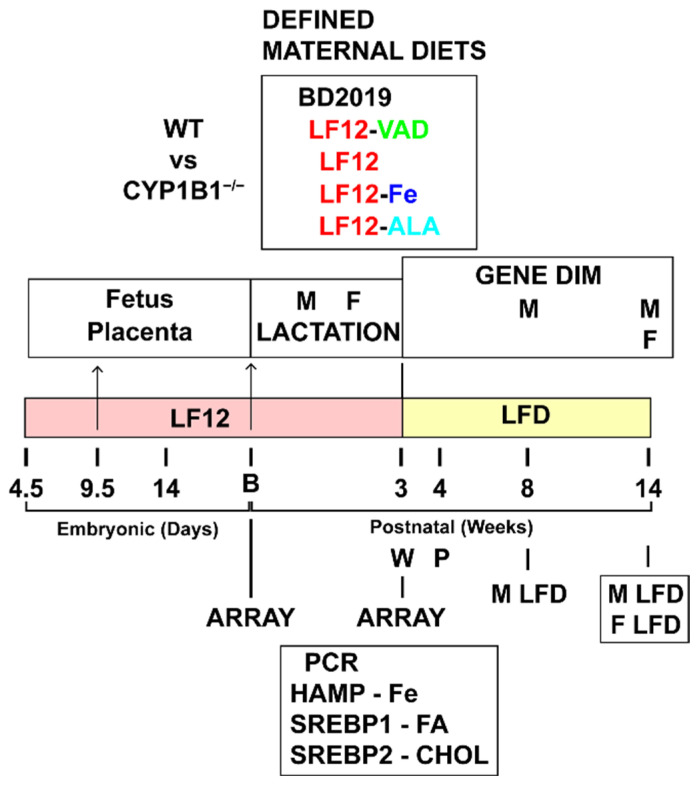
Experimental design for testing the impact of defined breeder diets and *Cyp1b1* deletion on neonatal gene expression measured as mRNA levels. An optimized commercial soybean oil-based diet (BD2019 or BD) was used for breeding of nulliparous females. On day 4.5, LF12 cotton-seed oil-based diets deficient in ALA and lacking iron supplementation replaced the BD until P21 weaning. For all treatments, WT and *Cyp1b1^−/−^* C57BL/6J mice were used. LF12 was also supplemented with iron or ALA to BD levels or deprived of retinol (VAD). At birth, gene expression was measured for livers combined for a full litter at midday (P0.5). At P21, individual livers were characterized by specified mothers. For unbiased characterization of liver dimorphism, microarrays were assessed for male expression at week 8 and for female expression at week 14. *Cyp1b1^−/−^*/WT expression ratios were determined in males at week 14. Abbreviations—M: male, F: female, DIM: dimorphic, B: birth, W: weaning, P: puberty, FA: fatty acid, CHOL: cholesterol, VAD: vitamin A deficiency, ALA: α-linolenic acid, and LFD: low-fat diet. Key additional information on animals and diets coding is described in Section 4. Color coding used in the Tables is described in Section 4.5.

Previous work has suggested that perinatal Cyp1b1’s effects on liver development impact adult metabolism and that this intervention is highly dependent on the maternal diet. Optimized commercial breeder diets potentially mask adverse effects on development. Here, we compare the Tekland breeder diet (BD2019) to defined diets (LF12) that are introduced 4 days after the start of pregnancy. LF12 is limited in two major ways (Figure 1, Table S5). First, replacement of soybean oil (SBO) with cottonseed oil (CSO) removes ALA [45,48]. The basic LF12 diet contains the standard adult level of iron without the 3-fold pregnancy supplementation that is included in the BD2019 diet. Each of these decreases enhanced the effects of *Cyp1b1* deletion on growth and gene expression at weaning. This transition to the adult diet on postnatal day 21 (P21) precedes male puberty by 7 days. The additional treatments shown in Figure 1 compare the effects of retinol depletion from LF12 (LF12-VAD) on both WT and *Cyp1b1^−/−^* mice. This VAD version of the LF12 diet delivers an extreme depletion of retinol, retinoic acid, and retinyl esters from liver [34]. This VAD failed to overcome the homeostasis that controls serum retinol, which is sustained by binding to high levels of *Rbp*4 (retinol-binding protein 4). The liver retinoid depletion only becomes effective from about four days before birth [8]. Mice were sacrificed for liver isolation at birth and P21.

We also compared the effects of LF12 supplementations with, respectively, iron (LF12-Fe) and ALA (LF12-ALA). For birth analyses, we combined livers from individual litters. Previous microarray analyses have established comprehensive suppressions by *Cyp1b1* deletion as a result of *Srebp1* and *Srebp2* activities [27]. PCR analyses were performed at P21, focusing on seven Srebp markers together with *Hamp* forms 1 and 2. Whole litters were used at birth and individual mice at P21. Treatment responses derived from Microarray data have been systematically color coded. This data also uses the same LIMMA designations for Statistical differences and is summarized in Section 4.5.

### 2.2. Cyp1b1^−/−^ Effects on Liver Gene Expression at Birth

**Figure 2:**
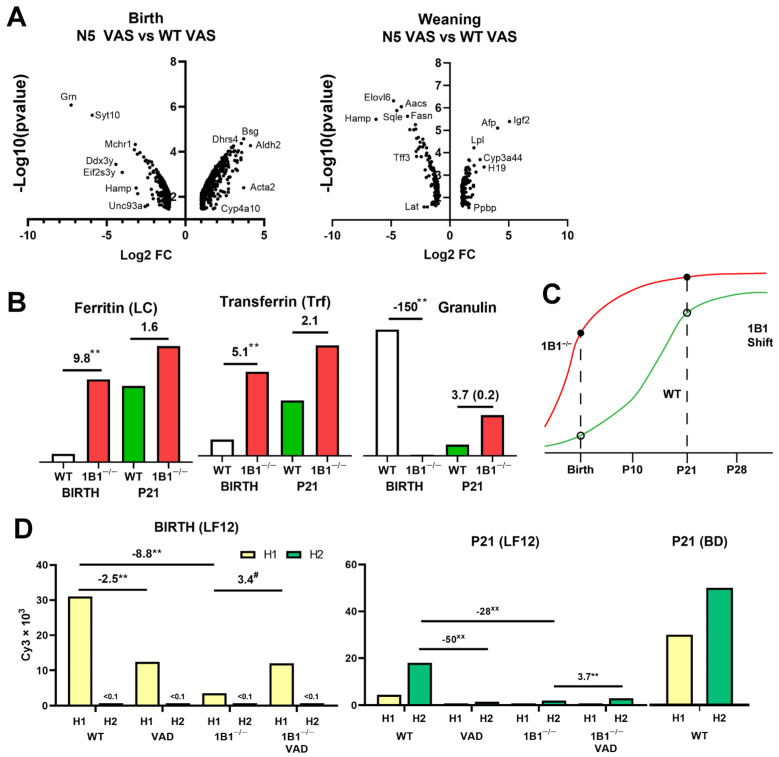
Birth and weaning *Cyp1b1^−/−^* changes compared to WT, each using LF12 defined diet. (**A**) Volcano plots log_2_ FC (fold change) versus Log_10_ *p*-value for N5 *(Cyp1b1^−/−^* backcrossed for 5 generations) at birth and weaning. (**B**) Birth and weaning expression for *Ftl1*, *Trf*, and granulin (*Cyp1b1^−/−^* vs. WT). (**C**) Model for high *Cyp1b1^−/−^* expression at birth that matches the WT weaning expression. (**D**) Effects of *Cyp1b1^−/−^* and VAD on *Hamp* (H1 and H2) expression at birth and weaning, showing the switch from Hamp1 to Hamp2 isoform in neonatal period. ^#^ *p* = 0.1; ** *p* < 0.01; ^xx^ *p* < 0.01.

The first goal was to test whether *Cyp1b1* deletion had distinct effects at birth and weaning (Figure 2A). Previous analyses of mice at birth and weaning showed that *Cyp1b1^−/−^* had no effect on the distribution of retinol and retinyl esters [34]. Volcano plots of microarray expression data show expression differences between WT and *Cyp1b1*^−/−^ effects at birth and p21 (Figure 2A). There were remarkably few overlaps in the effects of *Cyp1b1^−/−^.* Critically, the few VAD effects typically paralleled select effects of *Cyp1b1^−/−^*. *Cyp1b1*^−/−^ livers showed major stimulations of *Afp*, *Igf2* and *H19* that are associated with hepatoblasts [38,39].

A major feature at birth is the increased activation of HSCs marked in the Volcano plot by *Acta2* (encoding smooth muscle actin). Stimulations of *Acta2* and other HSC activation markers are shown in Table 1. The stimulations by VAD are equal to those by *Cyp1b1^−/−^*. They include collagen forms *Col1a1, Col1a2, and Col3a1*, an additional ECM protein fibulin 2 (*Fbl2*), and *Cyp2s1*, which metabolizes retinol.

Despite the dual effects on *Hamp*, *Cyp1b1^−/−^* caused major increases in transcripts for proteins that mediate iron regulation (Table 1). Unlike the parallel HSC activation, VAD had no effect on these genes either at birth or P21 on WT or *Cyp1b1^−/−^* livers. The serum iron transporter, transferrin (*Trf*), was highly stimulated. Transferrin mediates uptake through the transferrin receptor (*Tfrc*), which is not stimulated. This iron is then stored by the multi-subunit protein ferritin. The light-chain regulatory subunits (*Ftl1* and *Ftl2*) were each stimulated 9-fold.

*Trf*, *Ftl* forms, and *Hfe2* mRNA share the same pattern of stimulation, in which the increase in the birth of *Cyp1b1^−/−^* mice is matched by subsequent neonatal increases (Figure 2B). This pattern of regulation connects *Cyp1b1* stimulation at birth to elevated WT expression at P21. The major changes in liver development in the initial five postnatal days suggests that this increase occurs long before P21. For each gene, the WT stimulation models indicate that an advancement in *Cyp1b1^−/−^* occurs during or prior to an iron-dependent developmental transition (Figure 2C). This pattern of regulation was replicated by over 100 genes, many of which had associations with ferroptosis (*Gpx4*). *Fads1* is a notable inclusion, since this desaturase is critical for the synthesis of C20:4 arachidonic acid (AA), the precursor not only of prostaglandins and leukotrienes, but also of EETs [49].

The dominant ferritin heavy chain *Fth* is expressed at 20-fold higher levels but is unaffected. The product of smaller *Ftl* functions as a key regulator of ferroptosis [50,51]. *Hfe2* (hemojuvelin) is an activator of the hepatocyte bone morphogenic protein receptor (Bmpr2) for bone morphogenetic protein 6 (BMP6), the key stimulant of *Hamp* in hepatocytes. A smaller cluster dominated by highly expressed granulin (*Grn*) and *Syt10* (Synaptotagmin 10) appears in the Volcano plot (*Mchr1*, *Eif2s3y* and *Ddx3y*). Other highly expressed participants include *Rusc1* (Hh: Hedgehog regulator), *Klf2* (transcription factor and *Dusp8* (S/T kinase) (Table 1). This shift is the reverse of the *Cyp1b1*-dependent *Ftl* advancement. The transition from high *Cyp1b1^−/−^* expression at birth to low expression on P21 is accelerated. Granulin is an established neural growth regulator, and this large clustered response to *Cyp1b1^−/−^* may represent a response to strong granulin activity. *Dusp8* and the stem cell transcription factor *Klf2* are potential mediators of a granulin-mediated change. *Cyp1b1*-dependent granulin or associated genes may also restrain the *Cyp1b1*-dependent *Ftl*/*Trf*/*Hfe2* transition.

There was only one unifying feature at birth and weaning: extensive suppression of *Hamp*, which encodes the precursor of the 25-amino acid iron regulator hepcidin (HepC). The two *Hamp* forms are uniquely encoded at the same locus in mice. The hepcidins have 8 amino acid substitutions leading to differences in functional selectivity [28]. Interestingly, *Hamp2* is absent at birth but becomes the dominant form between birth and weaning. In the adult, at week 14, the two forms are equally expressed. *Hamp2* exhibits female liver dimorphism, which we show later is a selective feature of neonatal liver gene expression.

Each form of *Hamp* is suppressed by VAD and *Cyp1b1^−/−^* at both birth and weaning (Figure 2D). This suppression of *Hamp* at birth was completely prevented when *Cyp1b1^−/−^* mice were subjected to VAD. This treatment also removed the retinyl esters from *Cyp1b1^−/−^* HSCs but did not significantly affect the activation markers. This VAD reversal of *Cyp1b1^−/−^* effects was repeated in all *Srebp* suppression effects and in a set of functionally important gene stimulations. We suggest that this dual VAD-*Cyp1b1* signature marks the special HSCs’ involvement in neonatal liver development. The removal of HepC stabilizes the iron transporter Ferroportin (*Slc40A1*). The enhanced transport elevates serum iron that is available from macrophage turnover of erythrocytes and transport from the gut [28]. This change coordinates with the changes in iron transfer (*Trf*) and storage (*Ftl*). The *Hamp* restoration should also restore iron-level changes in *Cyp1b1^−/−^* mice.

### 2.3. H19 LncRNA Is a Potential Mediator of Changes Produced in Cyp1b1^−/−^ Mice

**Figure 3:**
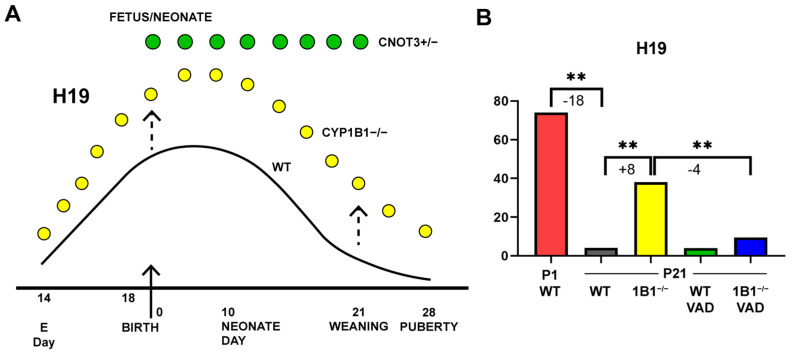
*H19* stimulations at birth and P21/weaning and additive with the underlying postnatal decline but is stimulated by the post retinol/stellate cell intervention. (**A**) Scheme for *H19* time course, birth to weaning. Stimulation by *Cyp1b1^−/−^* at birth matched the stimulation at P21, even though FC had increased from 1.7- to 8-fold. Regarding *Cnot^+^^/−^*, mice remain constant from birth to P21. (**B**) Effects of *Cyp1b1^−/−^* and VAD on *H19* at weaning/P21. Dual *Cyp1b1^−/−^*/VAD (Blue) blocks stimulation in *Cyp1b1^−/−^* with sufficient retinol (Yellow). ** *p* < 0.01.

Most of the 700 gene expression changes (>3-fold) that occur between birth and weaning remained unaffected in *Cyp1b1*^−/−^ mice. However, about 100 genes were substantially affected. *Igf*2, *Afp*, and *H19* lncRNA each reached peak expression 2 days before birth and then declined from about 5 days after birth (Figure 3A). For *Igf2* and *Afp*, less than 0.1% of the birth expression remained at weaning. The 20–30-fold stimulations in *Cyp1b1*^−/−^ mice at weaning represent only low proportions of the peak expression. *H19* retained 6% of the birth expression at weaning (Figure 3A,B). The 8-fold stimulation of *H19* in *Cyp1b1*^−/−^ mice at P21 maintained almost half of the WT birth expression.

*Igf*2, *Afp* and the *H19* lncRNA were not directly affected by VAD. Each stimulation, however, was highly suppressed when VAD and *Cyp1b1*^−/−^ were used together (Figure 3B). This reversal effect of the dual *Cyp1b1^−/−^*/VAD treatment is shared with *Srebp* suppression, which will be addressed in the next section. This reversal feature was shared by *S100a9* and most other weaning stimulations by *Cyp1b1^−/−^* (Table 2 and Table 3). *Krt23*, a stress marker, and the Histone *1h1b*, a chromatin modifier, are functionally important. We are attributing this very specific overlap of effects to HSC activity derived from the unique presence of stored retinol esters in these cells. In addition, *H19* in *Cyp1b1*^−/−^ pups was stimulated by 1.75-fold at birth (*p* = 0.02) (Table 3). However, this modest stimulation at the peak of expression generates a similar amount of additional activity as the 8-fold (Figure 3A).

The distinctive time courses for *H19*, *Igf*2, and *Afp* were completely changed in mice that were genetically modified to a hemizygous state for mRNA regulator *Cnot*3 (*Cnot*3^+/−^ mice) (Figure 3A) [40]. The characteristic neonatal decline was completely removed. *Cnot3* inactivates RNA by increasing de-adenylation and de-capping through the *CCR4*-NOT multiprotein complex. *Cnot*3 was decreased by *Cyp1b1^−/−^* at birth (Table 4). This change was matched by a decrease in *Pan*3, a key participant in a second contributing complex within this mRNA inactivation process [52].

**Figure 4:**
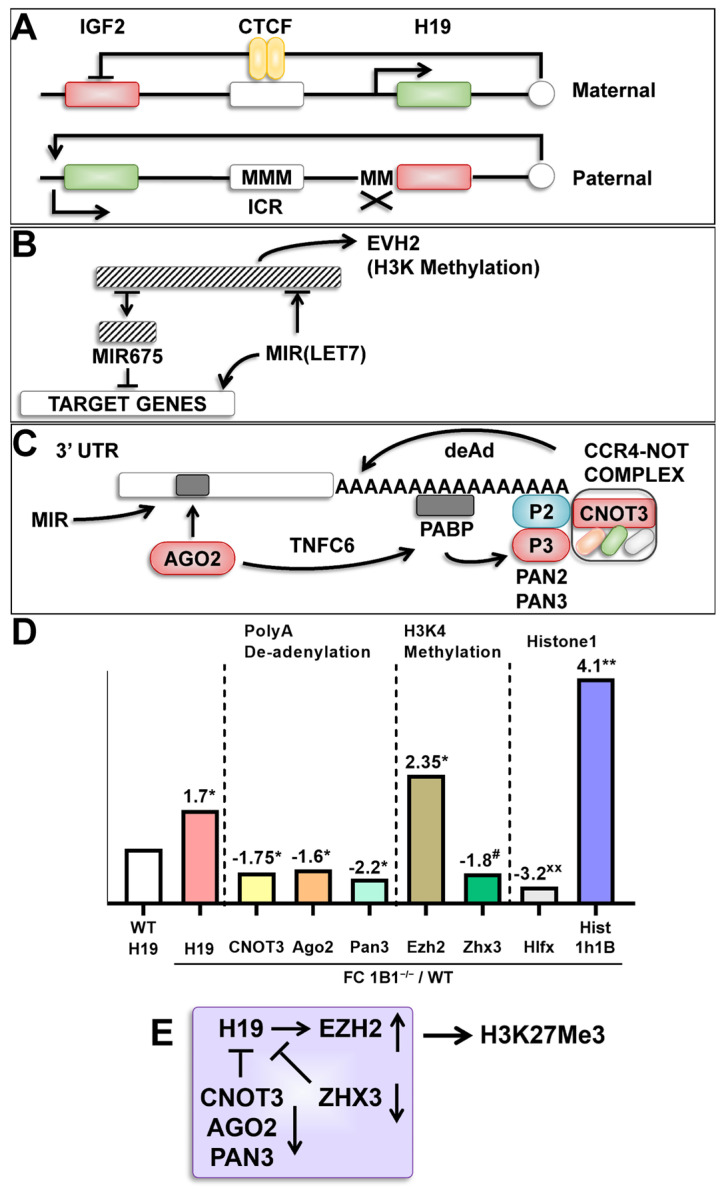
*Cyp1b1* deletion effects on *H19* are modulated perinatally by effects on *Ago2*, *Pan3*, and *Cnot3* and by chromatin effects of *Zhx3*, *Ezh2* and *Hist1h1B* (see Table 3). (**A**) *H19* LncRNA is expressed from the maternally derived unmethylated gene (green). The regulator CTCF assists the binding of *H19* to the open ICR element. *Igf2* expression from the maternal gene (red) is blocked by CTSF. On the paternal chromosome, *H19* and the ICR site are each methylated. CTCF does not bind; the *H19* gene is inactive but *Igf2* is transcribed. (**B**) Three mechanisms for H19 activity: miRNA-675 is cleaved to inhibit multiple target genes. *H19* also sequesters miRNA (notably *Lin7*) to enhance the transcription of targeted genes. *H19* binds to *Evh2*, a key mediator of H3 methylation. This histone modification is a common gene-suppression process. *Evh2* activity is inhibited by *Zhx3*, which is suppressed. (**C**) The miRNA inhibition is a prime regulatory process that is modulated by *H19*. *Ago2* mediates the miRNA suppression process by binding to the 7- or 8-base miRNA target sites in the 3′-UTR of susceptible genes. *Ago2* partners with *Tgfc6* and PolyA-binding protein (PABP) to recruit two de-adenylation complexes: *Pan2*/*Pan3* and the Ccr4-Not multiprotein complex. *Cnot3* is a key component of the *Ccr4-Not* complex that inactivates mRNA through de-adenylation and de-capping. (**D**) Stimulations of *H19* and *Evh2* matched by suppressions of modulators *Ago2*, *Cnot3*, *Pan3*, and *Zhx3*. (**E**) *H19* connects to chromatin gene suppression through generation of gene-selective H3K27Me3. ^#^ *p* = 0.1; * *p* < 0.05; ** *p* < 0.01; ^xx^ *p* < 0.01.

*H19* as a signaling lncRNA is only expressed from the maternal DNA at the *Igf2* locus (Figure 4A,B). *Igf2* is expressed from paternal DNA. Both are under the control of CCCTC-binding factor (CTCF) according to DNA methylation at the imprinting control region (ICR) DNA which is located between these two genes. *Igf2* is the prime controller of early neonatal liver growth, though a switch to *Igf1* occurs after weaning. This *H19*/*Igf2* regulatory center delivers epigenetic information from maternal and paternal sources to the offspring. *H19* has been linked to multiple miRNAs regulatory processes [52]. The miRNAs function through hybridization with targeted genes. The resulting complex is recognized by *Ago2*, which, with *Tnfc6*, links to PolA-binding protein (PABP) (Figure 4C). The chain continues with recruitment of CCR4-NOT and PAN complexes that initiate de-adenylation and de-capping to remove the target mRNA.

*H19* functions in several processes to release miRNA-675, including removal of *let7* [39]. Each has a select cluster of target genes that are, respectively, inhibited or stimulated by *H19*. Figure 4D assembles the set of *H19*-associated epigenetic modulation responses. *Ago2*, *Cnot3*, and *Pan3* mediate the inhibitory activities of miRNA. *H19* functions in a third way by directly binding the catalytic subunit of the polycomb PCR2 assembly to Ezh2. The *Ezh2* methylase produces H3K27Me3 epigenetic suppression. This suppression is central to growth hormone (GH) dimorphism [43] and to mTORC1 activity [53]. *Ezh*2 is stimulated in *Cyp1b1*^−/−^ livers to a similar extent as *H19*. *Cdk4*, a stimulant of *Ezh2* that is highly elevated, along with *Ap2m1*, which re-engages senescent cells [54]. *Zhx3*, an established repressor of *Ezh2*, is suppressed by *Cyp1b1*^−/−^. The changes in *H19* produced by *Cyp1b1* and retinol are likely to emerge from HSC/EC crosstalk.

*Cyp1b1*^−/−^ may also intervene by reorganizing Histone1-associated linker histones. Figure 4D pairs *H1fx*, which decreases, with *Hist1h1b*, which increases extensively [55]. *Hist1h1b* shares the *Cyp1b1*/VAD response of *H19* (Table 2), suggesting some level of co-regulation. Figure 4E summarizes an *H19*/*Ezh2* network that connects to gene suppression by H3K27Me3.

### 2.4. Other Stimulations in Cyp1b1^−/−^ with Preferential Expression at Birth

The application of comprehensive microarray analyses delivers an unbiased perspective on the specificity of the *Cyp1b1* interventions. The *Cyp1b1^−/−^*/VAD signature extends not only to *H19* but also to very highly expressed *S100a9* and *S100a8*. They are best known as calcium-binding proteins that function as heterodimers in neutrophils. At birth, the presence of a large number of monocytes was indicated by high expression of *Lyz*2. This monocyte lineage marker declined 5-fold at weaning but *S100a9* declined far more, which was indicative of a specialized subpopulation. *S100a9* is among the most highly expressed genes at birth. This duo is expressed here in specialized myeloid-derived suppressor cells (MDSCs) which inhibit T cells. *S100a9* is essential to the induction of MDSCs by lactoferrin in the maternal milk [56]. Identification of the time course of change is critical. The linker H1 histones, *Krt*23, Glypican3 (*Gpc*3) and *Igfbp*2, which decline from birth to weaning, each showed selective stimulation in *Cyp1b1*^−/−^ mice at weaning that was reversed by VAD.

### 2.5. Suppression of Srebp Regulation by Cyp1b1^−/−^ and VAD

Over 30 gene expression suppressions by *Cyp1b1*^−/−^ exceeded 3.5-fold with domination by genes that are associated with regulation by *Srebp1* and *Srebp*2 [8,33,57]. VAD suppressions were essentially all found among genes that are also suppressed by *Cyp1b1*^−/−^ (Table 4 and Table S1). This cluster included nine genes associated with fatty acid synthesis that are regulated by *Srebp1*. *Srebp*2 controls all steps in the cholesterol synthesis pathway. All Srebp2-mediated steps were extensively suppressed by *Cyp1b1*^−/−^. *Srebp2* regulation was distinguished from *Srebp1* regulation by resistance to VAD. The *Cyp1b1^−/−^* response genes that were unaffected by VAD were all linked to *Srebp2* involving cholesterol synthesis [57] or trafficking [58,59,60].

**Figure 5:**
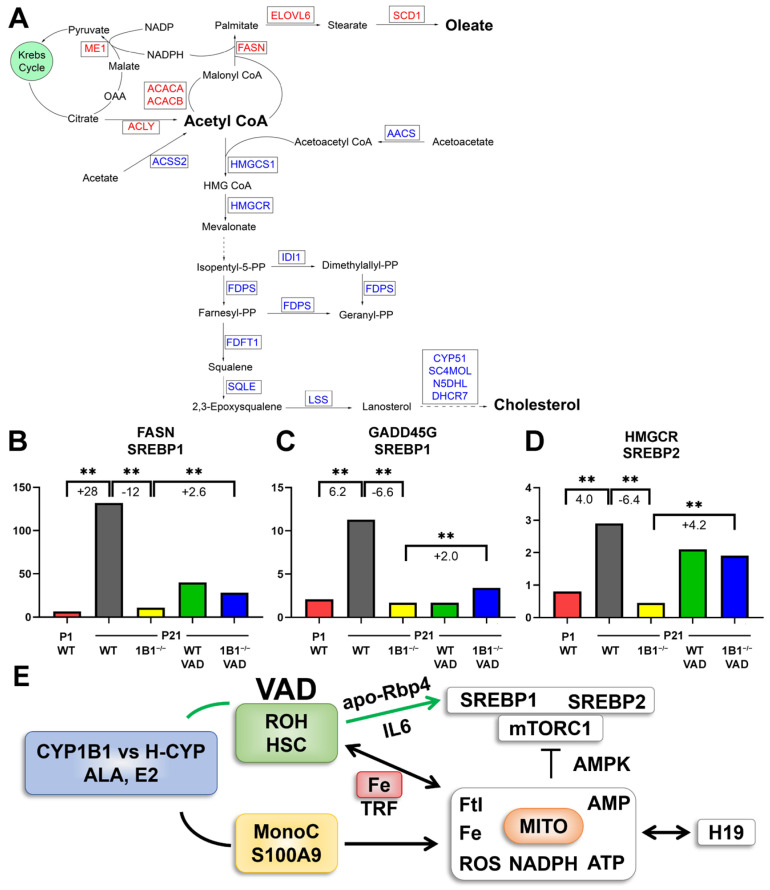
Effects of Cyp1b1 deletion and VAD on Coordinate regulation of fatty acids and cholesterol by Srebp forms. (**A**) Pathways for fatty acid and cholesterol synthesis, which are mediated by *Srebp1c* and *Srebp2*, respectively. (**B**–**D**) Effects of *Cyp1b1^−/−^*, VAD, and their combination on *Srebp1* marker *Fasn*, co-expressed *Gadd45g* and *Srebp2* mediated Hmg-CoA reductase. (**E**) Model of stellates link to intra-hepatocyte iron control processes. ** *p* < 0.01.

In Figure 5A, we show the full pathways from Acetyl-CoA, the shared precursor to the final products: respectively, oleoyl-CoA and cholesterol. These products are converted to cholesterol esters that are stored in lipid droplets that have a specialized membrane surface. Their synthesis is catalyzed by sterol-O-acyl transferase (*Acat*/*Soat* forms 1 and 2) [61]. *Acat1* is the dominant form at birth but is probably expressed in Kupfer cells, which are the preferred source. However, *Acat2* becomes almost equal through 10-fold stimulation by *Cyp1b1^−/−^* (Table 1). *Acat2* is favored later in neonatal development, matching the same pattern of *Trf* and *Ftl* forms (Figure 2B). The later availability of *Srebp* activity that controls the *Acat* substrates is consistent with the later entry of *Acat2*.

All these *Srebp*-regulated genes share a reverse version of the *H19* VAD signature that exhibits *Cyp1b1^−/−^* suppression instead of stimulation. The suppression in *Cyp1b1^−/−^* mice is attenuated when VAD is applied during their development. This signature is shown for *Fasn* and *Hmgcr* in Figure 5B,D. The *H19* signature is shown in Figure 3B. The *Cyp1b1^−/−^*/VAD signature for *Gadd45g*, a highly expressed regulator of cell senescence, is very similar to that established for *Fasn* (Figure 5C).

The rarity and specificity of the VAD intervention and two distinct types of crosstalk between VAD and *Cyp1b1^−/−^* implicate the HSCs. Both function for *Srebp1*, whereas only the *Cyp1b1* deletion reversal is involved for the *H19* stimulation group (Table 2, Figure 3) and the *Srebp2*–cholesterol regulation. HSCs link to *Srebp1* through stimulations of cytokines that increase *Stat3* via cytokine receptors on hepatocytes (Il-6/Il-6r) [62]. We hypothesized that HSCs link to a novel *Cyp1b1*-Fe control process that targets redox regulation and ferroptosis via transferrin/*Trf* and *Ftl* forms. Key elements for this redox unit include NADPH processing of ROS and mitochondrial generation of ATP that couples with both general energy metabolism and GTP-linked organelle fusion–fission cycling. Low energy is balanced by AMP kinase signaling. AMP kinase targets key proteins of the mTORC1 complex to suppress kinase activity [53]. mTORC1 kinase activity stimulates the essential activation cleavage of *Srebp* forms in the Golgi [53].

*Lpin1* [63], *Rdh11* [64], *Gadd45g* [65], four *Mup* isoforms [66,67,68] (major urinary protein 3) and *Rgs*16 [69,70,71] each fit the *Srebp*1 pattern (see color coding in Table 4 and Table S1). The similarity of the *Cyp1b1^−/−^*/VAD response pattern for *Fasn* and *Gadd45g* is shown in Figure 5B,C. Each gene retains the reversal response for the combination of VAD with *Cyp1b1*^−/−^ mice.

These genes each have distinctive functions with respect to liver development (Table 4). *Gadd45g* is notable for its modulation of embryonic stem cell activity [65]. *Lpin*1, which has been linked to *Srebp*1 activity, functions as a co-activator [63]. *Rdh*11 [64,72] is notable for reversing the normal dehydrogenation of retinol by *Rdh*10. Mups sequester circulating volatile organic metabolites and, when deleted by CRISPR editing, extensively shift the metabolism to an anabolic bias [67]. *Rgs*16 is a suppressor of the pro-inflammatory functions of monocytes [71]. A few genes outside the cholesterol synthesis pathway exhibit the *Srebp*2 pattern. Three such genes include *Pcsk*9 [58], *Stard*4 [59], and LDL receptor [60]; each is linked to cholesterol trafficking.

The *Cyp1b1*^−/−^ mice that result from prolonged maintenance and breeding practices involving the BD diet fail to suppress diet-induced obesity (DIO), i.e., they become resistant (R-*Cyp1b1*^−/−^, Table S2) [1]. Uniquely, two nuclear factors, *Dbp* [73] and *Dmbt*1 [74], respond almost as strongly to R- as they do to normal *Cyp1b1*^−/−^.

### 2.6. Use of Scd1 and Other Srebp Activity Markers to Compare BD and LF12 Diets

We performed separate tests to determine the effects of restoring the supplemental iron content and ALA to the LF12 diet. WT and *Cyp1b1*^−/−^ mice were compared for *Scd1* and four other genes: two that respond to *Srebp1c*, two that respond to *Srebp*2, and the two *Hamp* genes. In Table 5, we focus on comparing four of these expression markers across three diets (BD, LF12, and LF12-Fe). As noted earlier (Figure 2D), there was a switch from *Hamp1* at birth to *Hamp2* during the neonatal period. Male and female littermates provided similar expression levels (Tables S2 and S3). Their responses were therefore combined to provide mean expression levels for each litter.

**Table 5:** Effects of *Cyp1b1^−/−^* on multiple *Srebp1 and Srebp2* activities in relation to *Hamp* expression. (**A**) ΔCt Effects of *Cyp1b1* deletion (*d1b1*), including with iron supplementation on mean values for the multiple litters in each treatment (BW: Bodyweight). WT coded in **red** and *Cyp1b1^−/−^* coded in **black** difference (*d1b1*) is blue. (**B**) Individual litters separated into three response ranges ranked over all treatment groups (see ranges below). Ranking is color-coded across all treatments to probe expression diversity. The ranking of 24 litters is color-coded across all treatments into approx. equal ranking groups (7 to 10). BW/growth is also similarly ranked.

(**A**)						
**Litter**	BW	**Me1**	**Scd1**	**Elov6**	**Hmgcr**	**Hamp1**
		**Srebp1**	**Srebp1**	**Srebp1**	**Srebp2**	
**BD**	**8.7**	**2.9**	**−0.8**	**4.4**	**2.7**	**0.4**
**BD-*1b1***	**7.9**	**3.9**	**1.0**	**6.4**	**3.9**	**0.2**
***d1b1***	*0.8*	*1.0*	*1.8*	*2.0*	*1.2*	*0.2*
						
**LF12**	**7.5**	**2.6**	**−0.1**	**4.3**	**2.4**	**3.6**
**LF12-*1b1***	6.6	4.1	2.6	7.0	3.9	7.9
***d1b1***	*0.9*	*1.5*	*2.7*	*2.7*	*1.5*	*4.3*
						
**LF12-Fe**	**7.8**	**2.3**	**−1.6**	**2.7**	**3.7**	**2.3**
**LF12-Fe-*1b1***	6.7	4.0	0.9	4.6	3.4	1.8
***d1b1***	*1.1*	*1.7*	*2.5*	*1.9*	*nc*	*−0.5*
(**B**)						
	**BW**	**Me**	**Scd**	**Elov6**	**Hmgcr**	**Hamp1**
**BD**		−−−−−−	**Srebp1**	−−−−−−−	**Srebp2**	
**1**	10.2	2.4	**−0.8**	3.9	**2.9**	−1.5
**2**	9.0	2.2	−2.7	3.6	**3.5**	**3.1**
**3**	8.9	4.2	**1.6**	**5.5**	2.5	0.5
**4**	8.1	**3.0**	−1.3	3.9	2.6	0.35
**5**	7.2	**3.2**	**−0.5**	**5.2**	1.8	−0.4
**BD-*1b1***						
**1**	9.2	3.9	**−0.8**	**5.2**	**3.4**	**0.7**
**2**	7.8	3.9	2.0	7.0	4.6	**1.1**
**3**	6.8	3.9	1.9	7.0	**3.7**	−1.2
**LF12**						
**1**	8.7	1.0	−2.6	2.2	0.5	0.5
**2**	7.9	1.7	**−0.8**	2.0	1.7	**1.2**
**3**	**7.7**	**3.1**	**0.1**	**5.6**	1.6	**1.8**
**4**	7.5	**2.8**	−1.2	**4.4**	4.7	**3.3**
**5**	7.1	**2.6**	1.9	6.2	**2.5**	9.6
**6**	6.0	4.4	2.3	**5.5**	**3.3**	5.2
**LF12-*1b1***						
1	7.0	4.5	3.5	7.3	4.2	6.9
2	6.7	**3.2**	3.3	7.2	**3.6**	9.7
3	6.4	4.6	2.5	6.8	4.0	8.0
4	6.2	4.2	**1.3**	6.6	3.7	7.0
**LF12-Fe**						
1	9.1	0.8	−3.8	1.7	2.7	**1.6**
2	8.0	2.2	−2.9	1.2	**3.6**	**1.1**
3	6.4	4.0	2.0	**5.3**	4.7	4.2
**LF12-*1b1***-**Fe**						
1	7.7	**3.4**	**−0.2**	**4.2**	3.9	0.1
2	6.2	4.9	**1.6**	4.0	**3.4**	5.1
3	6.1	3.8	**1.4**	**5.6**	**2.9**	0.2

**Ranges:***Me1*1.0–2.6**2.7–3.9**4.0–5.8*Elovl6*1.2–4.04.1–5.65.7–8.9
*Hamp*−1.6–0.4**0.5–3.3**>3.3*Sqle*2.5–4.5**4.6–5.9**6.0–9.7
*Fasn*−0.1–1.6**1.7–3.0**3.1–5.6*Hmgcr*0.5–2.9**3.0–3.8**3.9–8.9
*Scd1*−3.8–−0.7**0.8–1.6**1.7–5.2Growth11.0-8.58.7–7.17.0–5.8

**Figure 6:**
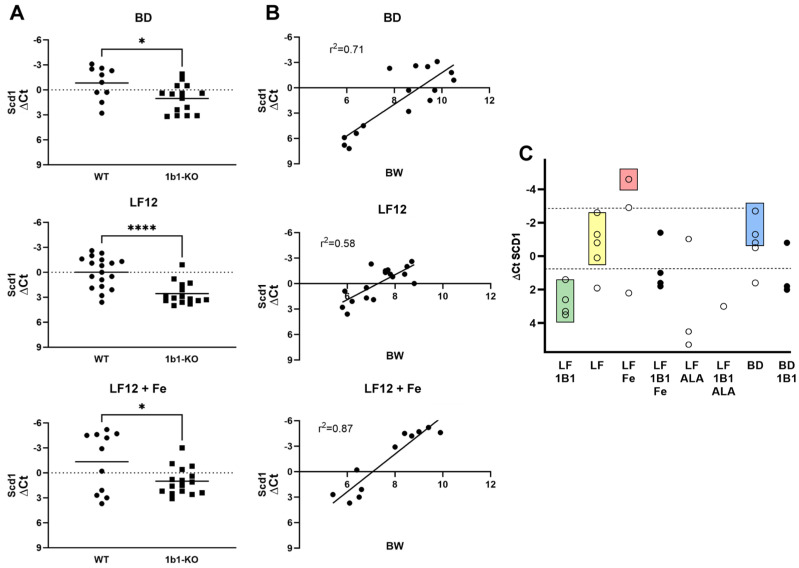
*Scd1* expression correlates with body weight (BW) for LF12 diets with slopes independent of iron and *Cyp1b1* but with different BW intercepts for respectively BD and LF12. (**A**) WT, versus *Cyp1b1^−/−^* for BD, LF12, and LF12-Fe. (**B**) Correlations of *Scd1* expression (ΔCt) versus BW for BD, LF12, and LF12-Fe. *WT* and *Cyp1b1*^−/−^ are included. (**C**) Displays *Scd1* expression for individual mice across 8 treatment groups. Colors indicate overlap with clusters identified by BW (Figure 7C). * *p* < 0.05; **** *p* < 0.0001.

The inter-litter differences were substantial. The mean expression levels for each marker and treatment group are shown in Table 5A; ΔCt increases represent expression decreases on a log2 scale. The ΔCt shift for *Cyp1b1^−/−^* is similar for each marker and is approximately doubled for LF12 compared to BD. The LF12 shift corresponds to 3- to 6-fold suppression, which is about half that shown in Table 4 for a separate microarray study. This difference is within the 10-fold range seen across different litters on the LF12 diet that correlate with BW (Table 5B, Figure 6B). Having a basis for such large differences is evidently important.

Table 5A shows that the expressions of the three *Srebp1* markers (*Me1*, *Scd1* and *Elovl6*) and the *Srebp1* marker (*Hmgcr*) are similar for BD and LF12 diets. The change from WT to *Cyp1b1^−/−^* increases consistently, but by less than 2-fold for all seven markers (Table S3). *Hamp1* and *Hamp2* differed significantly from the *Srebp* markers. *Hamp* forms are much more highly expressed with BD and relatively insensitive to *Cyp1b1^−/−^* with regard to this diet. *Hamp1* is far more highly suppressed in *Cyp1b1^−/−^* pups on LF12 than the *Srebp* markers. Again, this confirms the microarray data shown in Table 4. *Hamp2* is less sensitive to *Cyp1b1^−/−^* suppression than *Hamp1* (Figure 2D).

In Table 5B, this *Scd1* response spread for all six treatments has been ranked (color coded) for each litter across all treatments. This ranking is compared to the rankings of similarly regulated genes, elongase (*Elovl6*) and malate enzyme (*Me1*). Each one is regulated by *Srebp1* and suppressed by both *Cyp1b1^−/−^* and VAD (Table 4). Two other *Srebp1* markers, *Acss2* and *Fasn*, are used in an expanded comparison (Table S4). *Hmg-CoA* reductase from the cholesterol synthesis pathway is regulated by *Srebp2*. *Sqle*, which appears later in the pathway, is similarly ranked (Table S1). *Hamp1* is included as a marker of iron availability. *Hamp2*, which is more highly expressed in neonatal liver at P21 (Figure 2D), is somewhat less responsive (Table S4). *Acss2* closely matches *Me1* and is similar to but distinct from *Scd1*, particularly in terms of iron sensitivity.

The heterogeneity of LF12 litters is correlated within a single treatment group across their BW. The *Srebp* regulation and *Hamp* expression are indicative of fundamental developmentally set differences in regulation. The balance of yellow (high), red (intermediate), and green (normal) across the three *Srebp* markers (seven in Table S4) provides a refined perspective of the regulatory shift. The *Hamp* activity is sometimes parallel (coupled) and sometimes unrelated (uncoupled). The equivalent coding for BW (yellow, grey, and green) provides an equivalent perspective on growth. This may represent a visualization of the mTorc1 activity [63]. This multiprotein complex regulates lipogenesis through activation of *Srebp* forms versus growth, which is derived from amino acid recognition and associated protein synthesis stimulation.

*Cyp1b1^−/−^* was uniformly effective in suppressing *Hamp* and *Srebp* activities in LF12 litters (Table 5B). The diversity is shown for *Scd1* expression across all treatments in Figure 6C. The six WT LF12 litters included one that matched this level of *Cyp1b1^−/−^* suppression. Only one reached the lower margin of BD litters. Iron supplementation increased the diversity of litters. One litter reached the mid-BD range, one stayed in the mid-range, and one fell to the LF12-1B1 range. However, a third litter matched the suppressed *Cyp1b1^−/−^* litters in terms of *Srebp* activity. The 3-fold iron elevation had surprisingly little impact on LF12 levels of *Hamp* expression.

This matching of *Srebp* activities to BW based on color coding suggested that similar liver states may reappear across multiple treatments. *Scd*1 is the most highly expressed *Srebp* marker. The combined expression levels used in Table 5 are compared in Figure 5A. There was no significant effect for BD but clear significance for LF12 (Figure 6A). Iron supplementation lost this significance by spreading the responses into separate groups.

Sampling of multiple litters showed that expression levels for BD and LF12 diets each correlated with weaning BW (Figure 6B). Critically, combination BD and LF12 plots are appreciably displaced, although they converge at the lower BW. The center of the combination LF12 plot corresponds to higher expression and lower BW than that of the BD plot (ΔCt − 1.5, BW 7.8 g versus ΔCt + 2, BW 8.5 g). Iron supplementation of LF12 shifted BW into the BD range but did not affect the slope of the plot. We have addressed whether ALA causes this shift.

The distribution of *Scd1* expression across the different treatments (Figure 6C) provides a broader perspective on features identified with the correlation plot. The key points are as follows: (a) BD and LF12 diets each exhibited clustering with similar WT expression; (b) *Cyp1b1^−/−^* mice fed the LF12 diet exhibited a distinct lower cluster. BD mice only showed partial cluster separation for *Cyp1b1^−/−^* mice; (c) iron and ALA supplementations of LF12 provided stimulatory promotions, but only in select mothers; (d) iron only partially reversed the suppression in *Cyp1b1^−/−^* mice; and (e) other *Srebp1* markers (*Me1* and *Elovl6*) and the *Srebp2* marker (*Hmgcr*) showed similar distribution features (Table 5B). However, *Acss2* and *Me1* (Table S3) were insensitive to this iron supplementation in either WT or *Cyp1b1^−/−^* mice.

### 2.7. Resolution of LF12 Supplementation by Iron and ALA into Three Levels of Growth Control

**Figure 7:**
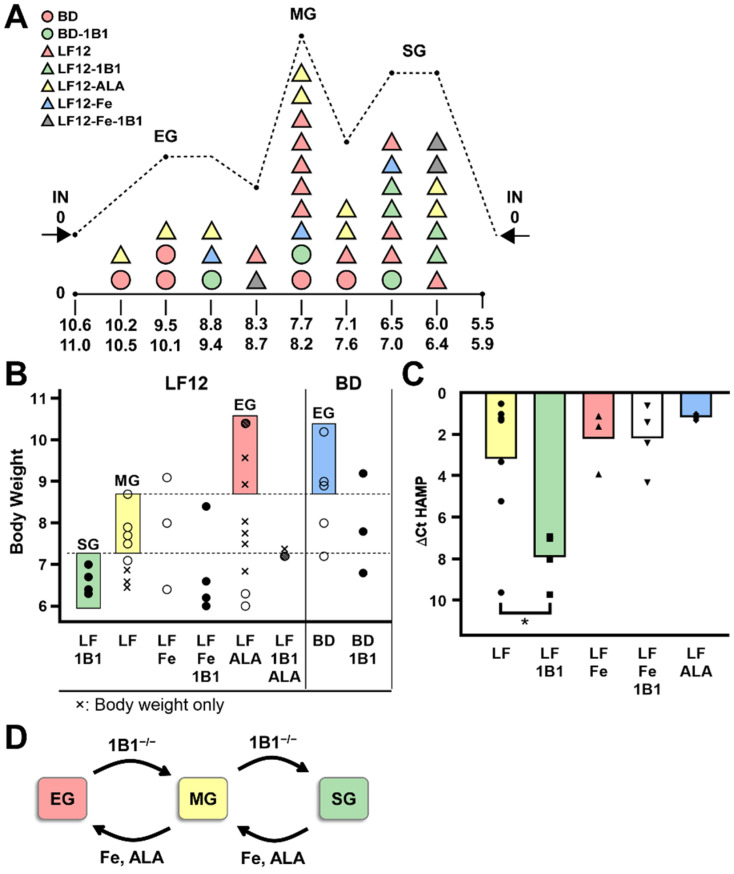
Three-state model for maternal support of embryos and neonates that engages the effects of iron, ALA and Cyp1b1 deletion. (**A**) 38 litters from 7 treatment groups were distributed across 10 BW increments. This distribution divides into three growth response groups: EG, MG, and SG. The outer groups had representation from single mice that weighed as much as 11.2 g or as little as 5.8 g. EG (8.8–11 g) is represented in 8 litters receiving 4 treatments. MG (7.1–8.7 g) is represented in 15 litters receiving all 7 treatments; SG is represented in 14 litters receiving 6 treatments. ALA and Cyp1b1 are prime effectors of distribution through opposing effects on the growth effects of Srebp. ALA supplementation of LF12 promotes LF12 to distribute over each growth while Cyp1b1deletion demotes BD into lower growth groups. The dashed line is displaced to show the distribution of increment numbers (IN). (**B**) Clusters captured from four treatments. Two treatments (LF-Fe and BD-1B1) have a single representation in each cluster. (**C**) Distribution of *Hamp*/HepC expression across five LF12 diets. Note that BD and BD-1B1 match LF-ALA’s high clustering. (**D**) Three-state model. *Cyp1b1*^−/−^ increased the probability that the litter will select MG or SG. Fe and ALA supplements shift the litter distribution in the opposite direction, although they achieve this via different mechanisms. The association between ALA and *Cyp1b1* is notable. * *p* < 0.05.

Additional P21 BW data were available from litters that were used for adult analyses (Figure 7A, Table S4). The mice from all four BD and LF12 diets with both WT and *Cyp1b1^−/−^* mice were distributed into ten BW increments (Figure 7B). These 40 litters were distributed into three size groups, each representing multiple treatment groups. We also displayed BW according to the treatment group (Figure 7C). For LF12 diets, there was an evident overlap with the *Scd1* display, confirming the linkage between *Srebp1* activity and growth. However, clustering at three BW levels is now better resolved for LF12-1B1 (low), LF12 (middle), and LF12-ALA (high) growth, respectively.

The comparison of the respective *Scd1* and BW displays (Figure 6C versus Figure 7C) is complicated by the relative downward shift in expression of BD relative to LF12 treatments. This displacement is also shown for the BD and LF12 correlation plots (Figure 6B). In effect, this shift means that the BD diet achieves much more growth relative to *Srebp* activities. Importantly, the shift was seen when addition of ALA to the LF12 diet caused an increase in BW to match the BD effects. This shift in the growth/*Srebp* relationship does not occur when iron supplementation of LF12 achieves a similar growth promotion. ALA evidently makes the LF12 diet more like the BD diet with respect to the growth/*Srebp* relationship. Iron supplementation promotes *Srebp* and growth. This occurs when *Hamp*/Hepc is coupled with growth. This lack of coupling is a major feature of the ALA route. The preliminary observation is that the shifts with each diet only occur at about one-third of the time.

Deletion of *Cyp1b1* is highly likely to steer the development into a low-growth/low-*Srebp* development route. This occurs most readily with LF12 when ALA is very low, and, conversely, less effectively in the BD diet when ALA is high. We propose that at least three different mechanisms are involved, each with low dependence on Cyp1b1 (*Cyp1b1^−/−^* litters), ALA (LF12 diets) and with high iron without *Cyp1b1* or ALA (LF12-Fe-1B1). This third process was seen when appreciable growth and *Srebp* activity were seen with high *Hamp* with the LF12-Fe-1B1 combination. The combination of LF12-ALA with *Cyp1b1^−/−^* produced moderate growth with very low *Srebp1* activity for two litters (not complete). Under these limiting conditions, we have data to support three options based on removal of *Cyp1b1*, iron, or ALA. The outcomes function in a probabilistic manner that depends on the mother.

This neonatal development may derive from earlier growth diversity that occurs with constant development progress (somite number) [8]. In a study on LF12 diet (E9.5), some embryos were over 2-fold smaller mid-gestation. This growth difference appears to derive from the mother, since growth equalizes on a post-weaning low-fat diet.

Based on the distribution data (Figure 7A) and the *Srebp*/*Hamp* data for individual litters (Table 5B), the developing fetus in each mother adopts one of three growth programs. The outcome depends on nutrient availability and maternal regulatory features: elevated growth (**EG:** 8.8–11.0 g), medium growth (**MG:** 7.1–8.7 g), and survival growth (**SG:** 5.5–7.0 g). Each program is distributed around the widely separated mean BW (9.2 g, 7.7 g, and 6.5 g).

Table 6 highlights distinctive features of the intervention from ALA supplementation. For each *Srebp1* activity marker, the EG BW/growth range is maintained on the BD diet with much lower *Srebp1* activity than for the LF12-Fe combination (Figure 6B,C and Figure 7C). Here, we show that for litters in the EG BW range (BD, BD-1B1, and LF12-ALA) these diets show very similar expression of each *Srebp1* marker. The litter on the LF12-Fe diet in the same size range has 6-fold higher *Srebp* expression (Mean ΔCt − 3.0, blue highlight). A similar 7-fold difference in *Srebp1* activity between BD and LF12 diets extended to the MG growth range for several different treatment groups. However, no LF12-ALA litters were available for marker analysis. In the SG range, there was no equivalent shift. Overall, the LF12-ALA diet shifts the expression of the LF12 diet strongly towards the more efficient BD support of EG growth. This 6–7-fold shift in EG and MG groups, which disappears in SG, derives from ALA.

Assessment of this distinctive ALA shift depends on the one LF12-Fe litter. This litter includes five mice with closely matched data (Table S3). Two further LF12-ALA litters out of ten have BW in the EG range (Table S3, Figure 7A). However, further analysis of ALA/EG litters is needed.

*Hamp* expression is much more susceptible to change in LF12 than in BD diets (Table 6, Figure 7D). The ranking of *Srebp1* markers for WT and *Cyp1b1*^−/−^ treatments with either LF12 or LF12-Fe diets matched the changes in Hamp (Table 5B). The match was extended for the additional markers (Table S4). There was no match for either BD or the LF12-ALA combination. *Cyp1b1^−/−^* shifted all litters into extreme *Hamp* suppression (Figure 7C). For the LF12, LF12-Fe, and LF12-Fe-1B1 litters, the high suppression outliers each have maternal deficiencies that caused the offspring to exhibit SG characteristics.

For all BD and BD-1B1 litters, the *Hamp* expression is high irrespective of changes in *Srebp1* activity or BW (Table 5B and Table 6). The assignments to MG or SG states were uncoupled from *Hamp*. This same uncoupling was shown for LF12-ALA. Thus, ALA is most likely the factor in the BD diet that uncouples *Srebp* from *Hamp*. In contrast, for the LF12 diet, in the absence of ALA, *Hamp* and *Srebp* markers shift together. The same absence is observed when *Srebp*/growth activities are suppressed. There does not appear to be a difference between situations in which *Cyp1b1* is deleted to lower the activity into the SG zone or those in which LF12/WT litters are suppressed to this extent.

### 2.8. Neonatal Liver Gene Expression Exhibits High Expression of Multiple Cyps

Cyp1b1 lowers E2 activity by removing E2 but also generates oxylipins and other active metabolites [47,75]. These *Cyp1b1* activities raise questions about the functions of the relatively high levels of *Cyps* that are expressed in the perinatal hepatocytes. The high levels of numerous *Cyps* will certainly deliver an oxylipin-rich environment that will be controlled by the high levels of epoxide hydrolase that we measured in the neonatal liver.

The timing and location of *Cyp1b1* activity appears to be critical. *Cyp1b1* may generate functional metabolites close to active receptors or channels. In contrast, the highly expressed *Cyps* probably generate more polar but less potent PPAR and CAR activators [47,76].

Out of a total of 102 full-length mouse *Cyps*, 40 are appreciably expressed in neonatal liver, often with appreciable differences between birth and weaning (Table 7). The direction of development is highly variable and presumably functionally significant. Thirteen forms showed adult sensitivity to *Cyp1b1* deletion with an almost total bias (11/13) towards suppression. However, there was no significant effect on these forms prior to weaning/puberty. Two minor *Cyp2d* forms (*Cyp2d12* and *Cyp2d22*) showed high stimulation in *Cyp1b1*^−/−^ pups at birth. Each is linked to the pattern of developmental advancement that was highlighted for *Trf* and *Ftl* forms (Table 1, Figure 2B,C). Other *Cyp2d* forms are far more highly expressed in the neonatal liver (Table 7).

The remarkable selectivity of neonatal *Cyp* expression and *Cyp1b1* sensitivity is clearly related to sexual dimorphism in their expression. This expression of dimorphism (DIM) is conferred in adult male mice by distinctive acute 4 h pulses of GH. DIM genes are adapted to increase (M-DIM) or decrease (F-DIM) in response to this specialized signal. Female mice deliver a relatively constant GH release that exerts the opposite effects on these two gene types. The Waxman laboratory has established that the male and female bias of select genes is established by changes in gene-associated histone 3 modification that provides access to binding of *Stat5b* and *Hnf4a* [43]. Here, we raise the question of whether DIM is established early in liver development, before puberty. The DIM of adult liver expression has been attributed to the testosterone-induced GH pulses that appear after puberty. However, direct testosterone effects are not resolved.

We developed an unbiased method to screen for DIM liver expression modeled on previous findings [43]. Males exhibit the maximum DIM change after 6–8 wk. In females DIM expression is constant from weaning to 14 weeks of age. We found that the *Cyp1b1* deletion effect developed in this 8–14-week period and is clearly connected to GH DIM [42]. The *Cyp1b1* expression sensitivity was shown to be male-specific in this 8–14-week period. Therefore, *Cyp1b1*^−/−^/WT expression ratios were assessed via microarray analyses at this age (Table 8). These trends are not restricted to *Cyps*.

We have examined the basis of the *Cyp1b1* sensitivity. The screen detected over 200 DIM genes from the array analyses of mice performed according to our BD/LF12 protocol (Figure 1). We limited the level of expression. A feature of the DIM process is that many of the genes are linked to lipophile-inducible nuclear receptors. Specific drug induction greatly expands the range of DIM genes [76].

Here, we focused on neonatal basal expression and the remarkable dominant presence of constitutive *Cyps*. Fourteen *Cyps* exhibited F-DIM bias in adults and four exhibited M-DIM bias. A feature of DIM is that F-DIM genes lose expression after weaning/puberty, while M-DIM genes gain expression [42,43,76]. This is shown in Table 8; column 5 vs. column 2. Most, but not all DIM genes are sensitive to *Cyp1b1* deletion (11/14 F-DIM; 2/4 M-DIM). The relationship is, however, unidirectional. For F-DIM, 11/14 genes are suppressed in male *Cyp1b1*^−/−^ mice; 2/4 M-DIM genes are stimulated. Notably, the *Cyp2c* F-DIM family is DIM but is not sensitive to *Cyp1b1* change. This uncoupling is repeated for M-DIM *Cyp4a12* forms. In contrast, the M-DIM *Cyps* (*Cyp7b1* and *Cyp4a12b*) had lower expression at weaning. They only appeared in males after puberty and then were stimulated in *Cyp1b1*^−/−^ mice.

The *Cyp* response patterns are commonly conserved within sub-families that are also regulated by a distinct lipophilic nuclear receptor (CAR/*Cyp2b*; *Cyp3a*/PXR; *Cyp4a*/PPARα) [76]. *Cyp* forms in the 2d, 2e, 2f and 2j sub-families (Table 7) are highly expressed, but are neither DIM nor sensitive to *Cyp1b1* deletion.

The remarkable DIM of the *Cyps* and other markers has established that the neonatal liver is highly polarized towards female signaling. In Table 9, we focus on 21 representative DIM genes (9 *Cyps*; 12 non-*Cyps*; 8 M-DIM, and 13 F-DIM). We compare male expression at three ages (birth, P21/weaning, and week 8) to female expression at week 14. For F-DIM genes, the male expression at weaning matched the expression in females at 14 weeks (Table 9; green). For most M-DIM genes, the optimum 8-week expression was >30-fold higher than the weaning expression. However, for *Cyp4a12b*, the difference is only 4.5-fold, despite a male/female expression ratio of 6.9 (Table 8 column 3; Table 9 column 3). The lessening of this ratio derives from significant additive non-DIM regulation.

These male pups at weaning/P21 selectively express F-DIM markers but not M-DIM markers. They have acquired and retained a female state. Half of the F-DIM markers are fully expressed at birth, while half acquire this polarization during neonatal development. Interestingly, anomalies occur for the two female DIM markers, *Fmo3* and *Xist*, which exhibit time specificity, respectively, absent at birth or weaning. This perinatal polarization probably derives from stimulation by the maternal estradiol. Our analyses of Srebp activity markers and BW in male and female littermates showed no significant difference.

Among these dimorphic genes, only the F-DIM genes are expressed in neonatal liver (Table 9). Among F-DIM *Cyps*, half showed their peak expression at birth while the remainder retained appreciable expression. The M-DIM/F-DIM bias in adult mice derives from adaptation to post-pubertal GH pulses. This earlier perinatal F-DIM bias may be estradiol-derived. This striking bias of the perinatal liver development is consistent with the estradiol promotion of hepatobiliary fate decisions [24]. Such effects of E2 are also consistent with the role of E2 in the generation of HepC and in the key developmental functions of iron homeostasis.

## 3. Discussion

### 3.1. Cyp1b1 and Retinol Function Together to Target Liver Development Before Birth

A remarkable feature of these extensive effects of *Cyp1b1* is that they occur with scarcely detectable *Cyp1b1* in the liver. By contrast, there were near peak neonatal levels for forty hepatocyte *Cyps* (H-*Cyps*; Table 7). However, *Cyp1b1* impacts liver development in the fetus such that gene expression is extensively affected at birth (Figure 2, Table 1) [8]. A likely source for this impact is the high level of *Cyp1b1* in the ST-mesenchyme (STM). This tissue lies proximal to the developing heart and supports sinusoids and hepatoblasts from about E11.5. *Cyp1b1* also affects these sinusoids by controlling the oxygen sensitivity and adhesion processes that underpin vascular development [21,22]. A key finding in this report is that deletion of Cyp1b1 and depletion of retinol (VAD) each strongly activate stellate cells at birth while each also extensively suppresses Hamp, the gene that produces the iron regulator Hepcidin [28].

Lineage tracing analyses have established the progression of Stellate cells/HSC and perivascular mesenchymal support cells during early liver development [77]. Application of this approach to WT and *Cyp1b1*^−/−^ could show how these progenitor populations are affected by *Cyp1b1* during perinatal hepatocyte development. Examination of VAD effects at either birth or weaning showed remarkably few gene changes, most of which are matched by effects of *Cyp1b1* deletion. The 25-aminoacid iron regulator *Hepcidin* removes the key iron channel protein ferroportin thereby restricting iron availability [28]. *Cyp1b1* and retinol integrate their effects on Hepcidin through HSC. This route is strongly evident with the LF12 diet that was developed for VAD studies [27,34]. LF12 is deficient in ALA. A second route to homeostasis is directed through LSEC. This process depends on dietary ALA and predominates in mice bred on standard breeder diets such as BD2019 that contain extensive ALA. The differences between LF12 and BD diets are introduced in Table 5 and Figure 7.

**Figure 8:**
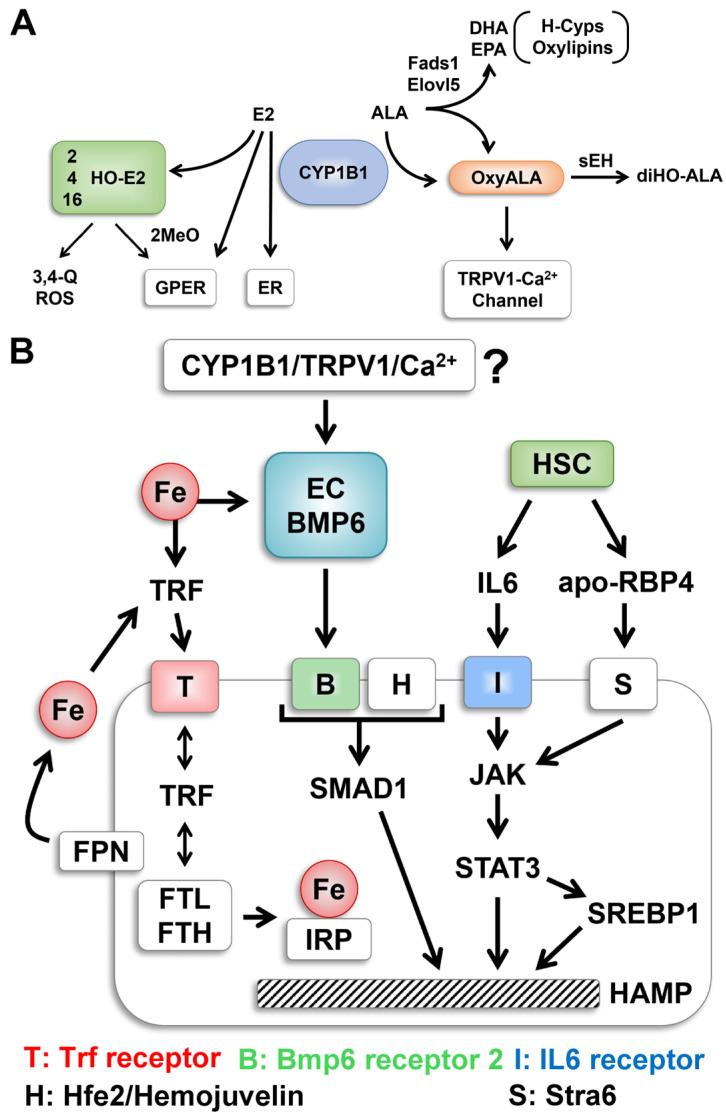
Cyp1b1 impacts cell signaling through metabolism of either estradiol (E2) or ALA. (**A**) E2 signals through nuclear receptors (ER) and membrane-bound G-protein-linked receptors (GPER). ALA is metabolized by Cyp1b1 to epoxide oxylipins that are further metabolized to diols (DiHO-ALA) by cytoplasmic soluble epoxide hydrolase (sEH), C-18 ALA is elongated by 2-carbons through Elongases (*Elovl5*) to C20 and C22 derivatives [79,84]. Desaturases *Fads1* and *Fads2* generate some C20 arachidonic acid (C20:4) but mostly C20:5 eicosapentaenoic acid (EPA) or C22:6 docosahexaenoic acid (DHA). These fatty acids are also substrate for Cyp1b1 or hepatocyte Cyps (H-Cyps). (**B**) Hamp transcription is activated by iron through endothelial (EC) generation of BMP6 that activates hepatocytes via BMP6 receptor (Bmpr2 partnered by hemojuvelin Hfe2). Smad forms deliver stimulation to the *Hamp* locus. An alternative activation via stellate cells that are directly affected by Cyp1b1 deletion and VAD deliver *IL6* and *apo-Rbp4* to respectively IL6 receptors or Stra6 to activate JAK/Stat3. *Srebp1* is a target of Stat3. *Srebp1* may share similar sensitivity to Cyp1b1 and VAD as lipogenic pathways. Iron homeostasis in hepatocytes shares distinct processing. Uptake through transferrin and transferrin receptors augmented by Hfe1 into the endosome network. Trf is recycled with release of iron to regulatory proteins (*IRP)*, activating *H*amp transcription to ferritin (*Ftl* and *Fth* subunits) for storage of iron and Ferroportin (*Fpn*) for exporting iron back to serum transferrin. Srebp activities were suppressed in Cyp1b1^−/−^ mice but with invariable reversal by VAD. The release of apo-Rbp4 from HSC and activation of Stra6 is probably responsible [32].

This process depends on the synthesis of BMP6 in LSEC generates Hamp with only minor effects of *Cyp1b1*.This process is enhanced by estradiol which is removed by Cyp1b1 [30]. ALA effects are typically mediated by oxylipins that are generated by Cyp1b1, multiple hepatic *Cyp* forms (H-*Cyp*) and other oxygenases and peroxidases [47,75,78,79]. These processes are discussed more extensively below with Figure 8.

A second VAD-*Cyp1b1* overlap occurs for the extensive elevation of *Srebp1* and *Srebp2* activities. *Srebp1* controls seven genes that synthesize C18-oleate from acetate (Figure 5A, Table 4 plus Supplementary Tables). Other *Srebp* regulated genes serve additional lipogenic functions. Included in this cluster is *Lpin1* which mediates the nuclear transfer and cleavage of *Srebp forms* [63]. There is scarcely any *Srebp1* activity at birth, but large increases occur between birth and P21 weaning. These increases are completely suppressed prevented in *Cyp1b1*^−/−^ mice when on the LF12 diet. On this diet VAD almost completely depleted stellate retinyl esters [27]. Srebp2 mediates about twenty steps in cholesterol biosynthesis. Each step was again almost completely suppressed in *Cyp1b1*^−/−^ neonatal mice. However, these *Srebp2*-mediated steps were uniformly unaffected by VAD. *Insig1*, which mediates feedback suppression of *Srebp* processes by cholesterol, follows the *Srebp2* pattern [58,59].

Significantly, the *Cyp1b1*^−/−^ suppressions of *Srebp1* and *Srebp2* were each extensively reversed by VAD treatment (Table 4). The selectivity of VAD effects suggests that the stimulations derive from *Cyp1b1*^−/−^ HSC. A feature of HSC when depleted of retinol is the synthesis and release of apo-Rbp4, a stimulant of Srebp forms in hepatocytes. The hepatocyte mediator is the Stra6 plasma membrane receptor which activates Srebp forms via Stat3 [32]. Crucially, *Srebp1* also stimulates transcription of *Hamp* [33]. This process is resolved from IL6 stimulation but each share Stat3 promotion.

This reversal mechanism extended to stimulations in *Cyp1b1*^−/−^ cells (Table 3, Figure 3B), including both the highly expressed regulator *LncrH19* and the Monocyte inflammatory Alarmin *S100a9.* These stimulations each correspond to an attenuation of the severe decline from high birth expressions (Table 3; Figure 3A,B). *H19*, *Igf2*, *Afp* and *S100a9* each share this decline. *H19* alone shows a nearly two-fold stimulation at birth without any corresponding effect on Srebp activities.

At weaning/P21, *H19* and *Srebp* activities are changed in opposite directions by *Cyp1b1* deletion. These effects are unlikely to be direct effects of *H19* on *Srebp* functions. Thus, release of exosomal *H19* from liver cholandiocytes activates *Srebp* forms. In addition, deletion of *H19* decreases *Srebp* activities and fatty liver [80,81]. An indirect coupling seems more likely. The neonatal liver development is associated with massive *Cyp1b1*-mediated increases in genes controled by *Srebp1* and *Srebp2* genes that correlate with liver growth (Figure 6). This growth is likely to be integrated particularly with the timing of differentiation steps. We show here that the promotion of liver growth on the BD diet wasmatched by lower *mTORC1* promotion of *Srebp* activities. A recent report [82] on neonatal mouse differentiation established that livers are fully formed at birth but with progress of features like lobe formation optimal with low *mTORC1* actvity. Slower growth (*Cyp1b1*^−/−^) may also slow the loss of markers of perinatal liver developmen such as *H19*, *Afp*, *Igf2* and *S100a9*. Nevertheless, stimulations from elevated *H19* increase may be important (Figure 4). The diverse miRNA signaling exhibited by *H19* is shared by the targeting of the *Cyp1b1* 3′-UTR via highly conserved miRNA processes [3].

*S100a9* typically marks the granulocyte lineage of monocytes that we have previously proven to be sensitive to *Cyp1b1* in bone marrow [1]. These cells neither express detectable *Cyp1b1* nor respond to deletion at birth. However, between birth and weaning, as these monocytes decline, a population that expresses *S100a9* is selectively sustained by 4-fold in *Cyp1b1*^−/−^ offspring. A novel myeloid lineage that controls the response to lactation has been identified in mice and humans [83]. A similar transient *S100a9* response has been characterized in the perinatal liver that is mediated by *Rab30* [56]. We see similar contributors (*Rab30*, *Fabp4*, *MT1*, and *MT2*) and preferential expression at birth.

### 3.2. The Integration of the Effects of Dietary Iron and ALA with the Activities of Cyp1b1 and Retinol

This exceptional sensitivity to *Cyp1b1* deletion only appeared after replacement of the optimized breeder diet (BD) with the LF12 retinoid depletion diet. LF12 lacks two key constituents: the usual pregnancy iron supplement and ALA. This C18 unsaturated fatty acid, is the prime dietary metabolic source for both ω − 3 C20:5 (EPA) and C22:6 (DHA) (Figure 8A). ω − 6 ∆4-Arachidonic is formed by the same enzyme sequence from linoleic acid which is abundant in both LF12 and BD (*Fads2*, *Elovl5*, and *Fads1*). Notably the ultimate step in these syntheses, *Fads1*, is stimulated 5-fold by *Cyp1b1*^−/−^ at birth (Table 1). Like *Ftl1* and *Trf*, *Fads1* is in the large cluster that is advanced in development by *Cyp1b1*^−/−^ (Figure 1c). *Elovl5* is not regulated by *Srebp1* but is much more highly expressed than any of these pathway genes. Oxylipins are generated from each of these polyunsaturated fatty acids (PUFA) through multiple diverse oxygenases, peroxidases, and P450 Cytochromes [47,75]. Notably, the P450 cytochromes including *Cyp1b1* generate epoxides (EET). EET retains the structural rigidity of the PUFA which generates high affinity binding but are rapidly removed by the highly expressed epoxide hydrolase, *Ephx2*.

**Figure 9:**
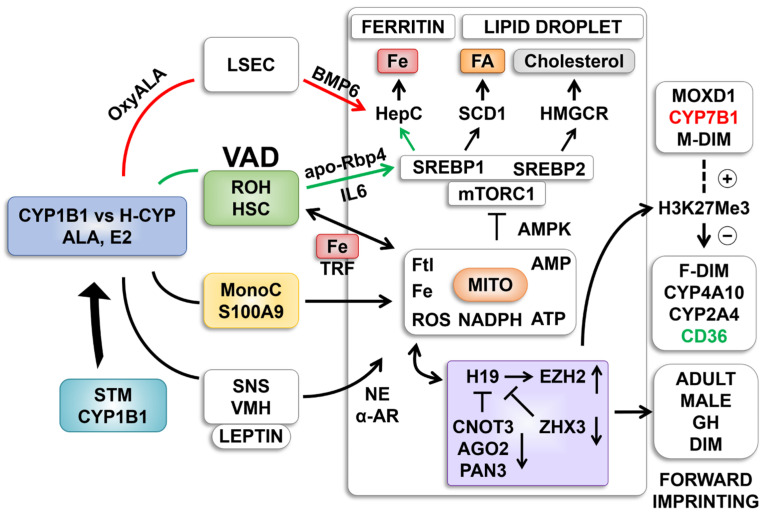
*Cyp1b1* affects hepatocytes through metabolism in cells that control four distinct external support processes. Four key sources of *Cyp1b1* intervention are LSEC, HSC, *S100a9*-specialized monocytes and the release of norepinephrine (NE) from leptin stimulation of the sympathetic nervous system (SNS) from the ventromedial hypothalamus (VMH). Septum transverse mesenchyme (STM) expresses high *Cyp1b1* at sites of liver initiation (E9.5). The relevant substrates include estradiol and ALA, which form OxyALA. Additional conversion to elongated unsaturated fatty acids, C20 EPA and C22 DHA, provide more oxylipins, including from hepatocyte *Cyps* (H-*CYPs*) (Figure 8). Communication between support cells and hepatocytes is poorly understood. Cytokines and chemical activators of hepatocyte receptors have been implicated in LSECs (Bmp6/Bmpr/Smad1), SNS (NE), and HSC (IL6 or apo-RBP). Monocytes most likely deliver cytokines and require *S100α9* participation. The responses within hepatocytes are mediated by select networks: *mTorc1*/*Srebp2*/HepC and *H19*/*Ezh2*/*Zhx3*/*Cnot3*. Two-way arrows indicate potential reverse effects from hepatocytes to support cells (HSC). A key question concerns the origin of oxylipins: *Cyp1b1* in support cells versus hepatocyte forms. Connections from the *Cyp* box (Blue) signify *Cyp1b1* activities within the connected cells. The effects of different diets (*Cyp1b1*^−/−^ impact) compared to LF12 or LF12-ALA diets (or BD) suggest differential effects of OxyALA and H-*Cyp* activity. The ALA effect is high in LSEC (promoting *Hepc* with low coupling with *Srebp*) and resistance to *Cyp1b1*^−/−^. OxyALA may then be derived from H-*Cyps*. Significant effects of *Cyp1b1*^−/−^ derive from HSC with low ALA impact.

In our current regulatory model two routes sustain Hamp with very different effects on Srebp activities. The dominant pathway with the BD diet is mediated by BMP6 in LSEC (Figure 9, red path) [30]. There is scarcely any loss from *Cyp1b1* deletion. The second pathway (Figure 9, green path) which predominates on LF12 depends on both *Cyp1b1* and retinol suggesting stellate cell involvement. *Stat3* mediated activation of *Srebp1* activates *Hamp* synthesis [33] (Figure 8B and Figure 9). IL6 from HSC can target the IL6 receptor on hepatocytes. At birth the strong suppression in *Cyp1b1*^−/−^ pups is reversed by VAD through the stimulation by apo-Rbp. Importantly, this LF12 sensitivity to *Cyp1b1* deletion and the strong linkage between *Hamp* and *Srebp1* markers are each removed by ALA supplementation of LF12. The BMP6 enhancement by ALA in endothelial cells could be directed from Oxylipin metabolites (H-*Cyp* or non-*Cyp* metabolism). Such metabolites mimic capsaicin in enhancing Trp channels [78]. Their transfer of Ca2+ activates diverse kinases. ALA involvement in the EC/BMP6 seems well supported but the mechanism needs to be addressed.

The diminished effect of *Cyp1b1* deletion suggests that the high levels of hepatocyte *Cyp* forms (H-*Cyp*) provide a likely source of Oxylipins (Table 7). Thirteen *Cyps* from 2, 3 and 4 subfamilies are highly expressed from birth to the mature adult (Table 8). Each is linked to *Hnf4a*, lipogenic receptors (PPAR, CAR and PXR) and their RXR partners [76]. Diverse Oxylipins activate these receptors. Each of these 13 *Cyps* has strong adult female expression bias (F-DIM) but retains near full expression at birth in male progeny (Table 9). *Cyps* or other genes with male bias (M-DIM) were not expressed. The F-DIM *Cyps* were highly suppressed in mature adult *Cyp1b1*^−/−^ males and yet were insensitive to *Cyp1b1* in the neonatal liver.

### 3.3. Evidence of HSC Cyp1b1 Participation in Liver Development

Since HSCs are the only major source of retinol esters, we propose that this unique set of responses derives from the sensitivity of these cells to *Cyp1b1*. Vascular expansion in the retina has been linked specifically to *Cyp1b1* in pericytes [85], which is particularly labile and linked to HSC adhesion [5]. The role of HSCs in liver fibrosis and NAFLD depends on their *Cyp1b1* activity [5]. The *Cyp1b1* expression and activity in these cells are labile and linked to cell adhesion. *Cyp1b1* also suppresses ROS and controls adhesion processes in an oxygen-dependent manner with links to *Hif1α*. Notably, the *Cyp1b1* promoter has a novel enhancer that includes *Ahr*/*Arnt* recognition elements suggestive of competition with *Hif1*/*Arnt* control [2].

A central feature of HSC activation is the abundant synthesis and release of adhesion proteins [86]. We showed that *Cyp1b1*^−/−^ and VAD each substantially activate HSCs, as evidenced by the stimulation of smooth muscle actin (*Acta2*) and a set of collagens (1α1,1α2 and 3α1) (Table 1). This burst of stimulation is not retained at P21, but the basal activation remains appreciable. This adhesion capacity stimulates the formation of vascular tubules in cell culture, which may model the development of liver sinusoids [21]. Recent research has shown that HSC populations are heterogeneous with respect to *Cyp1b1* content [46] but that the presence of *Cyp1b1* synthesizes ligands for growth regulatory receptors [6].

### 3.4. Evidence for a Central Role of LncRNA H19 in Cyp1b1 Signaling

This *H19* activity was linked to changes in regulatory factors and partners in the birth mRNA profiles. This data set from *Cyp1b1*^−/−^ mice shows that *H19* is 70% higher at birth and 7-fold higher at P21 (Table 2 and Table 3). These additional birth and p21 increments in *H19* are actually quantitatively similar (Figure 3A). This diagram presents the published finding that in *Cnot3*^+/−^ mice the postnatal decline of *H19*, *Igf2* and *Afp* is removed [38]. *Cnot3* is a component of the CCR4-NOT complex that destabilizes polyadenylated RNA through both de-adenylation and de-capping. *Cyp1b1* deletion is partially reproducing this Cnot3 effect. *Cyp1b1* deletion decreases *Cnot3* suggesting that the CCR-NOT complex is a key site of *Cyp1b1* intervention. This reversal implies that polyA RNA instability shown in the birth expression is central to the neonatal decline of *H19* and to the stimulation in *Cyp1b1*^−/−^ mice. Similar decreases were found for polA stability factor *Pan3* and for the *Ago2* the initiating mediator of miRNA suppression activities. These RNA manipulations are also matched by Histone 1 changes that direct chromatin structure (Table 2 and Table 3).

*Zhx3*, another nuclear regulator that suppresses *Afp and H19*, is also suppressed at birth. *H19* was not reported in the *Zhx3* studies, but the closely related zinc-finger transcription factor *Zhx2* regulates both *Afp* and *H19*. *Zhx3* is robustly expressed at birth, P21, and week 14 in males whereas Zhx2 was scarcely expressed. Zhx3 may therefore provide an equivalent contribution. *Zhx2* also mediates adult suppression of F-DIM Cyps (Table 7 and Table 8). Diminished *Zhx3* could contribute to the enhanced F-DIM polarization of the neonatal liver.

The *Cyp1b1*^−/−^ liver sustains *H19* and, similarly, regulates *S100α9*, which mediates a novel monocyte process that targets the interface between lactation and microbiome activities [83]. Other factors are implicated in a novel *Cyp1b1*/*H19* nutrient network including Glypican 3/*Gpc3* and the stress regulator Keratin 23/*Krt23*). *Afp* controls the availability and distribution of maternal estradiol, which is affected by *Cyp1b1* metabolism (Figure 8).

The multi-generational imprinting capacity is a key feature of maternal *H19* and paternal *Igf2* signaling. This is realized particularly in neuroendocrine signaling. For example, treatment of female mice with the glucocorticoid receptor agonist dexamethasone interferes with *H19* signaling in the adrenal gland. This altered *H19* activity in female offspring remains significant even after two further generations. This transgenerational signaling may contribute to the heterogeneity of maternal *Srebp* control described below [87].

### 3.5. Cyp1b1 Afftects a Diversity of Srebp Functions

Retinol is stored in specialized lipid droplets that are located exclusively in HSCs. *Srebp* regulation similar to that observed in hepatocytes also takes place in HSCs [86]. The magnitude of *Scd1* and *Fasn* expression implies that their control by *Cyp1b1* occurs in hepatocytes.

Outside the two mentioned pathways, the few targeted genes have potential linkage that is suggestive of unknown partnerships. *Rdh11*, which reverses the retinoid pathway back to retinol [64], exhibits the *Srebp1*/*Cyp1b1*/VAD signature. In this case, retinyl oleate esters and lipid droplets in stellate cells are affected. The *Srebp1*/*Cyp1b1*/VAD-mediated HSC linkages to *Gadd45g* stress regulation [65], *Rgs16*-mediated GTP signaling [71], and to *Mup* protein [67] transport of volatile metabolites are each rational contributors to a growth-development adaptation network.

Two key *Srebp* regulators are linked to the *Srebp1*/*Cyp1b1*/VAD signature. *Lpin1*, which mediates the cleavage activation of all *Srebp* forms [63], follows the *Srebp1* pattern. *Insig1*, which contributes to feedback suppression of *Srebp* processes by cholesterol, follows the *Srebp2* pattern [58,59]. The cholesterol transport functions of *Psk9* and *Stard4* are logical complements to cholesterol synthesis. Each follows the *Srebp2* pattern.

### 3.6. Role of mTorc1 in Srebp Signaling and Liver Growth

The twin *Srebp* controlled pathways in hepatocyte endoplasmic reticulum generates a store of cholesterol oleate the main constituent of lipid droplets. Dietary cholesterol esters are delivered from LDL via the endosome network that terminates in lysosomes that release cholesterol through a specific acidic lipase. The connection between these cholesterol sources has an important control over *Srebp* activities. This dietary cholesterol binds to the trans-membrane cholesterol transporter *Npc1* which transfers cholesterol to *Slc38a9* on the outside of the endosome. The cholesterol-Slc38a9 complex recruits a multiprotein complex; the mTORC1 complex [88,89]. This lysosomal binding is also mediated by a set of specific amino acid sensors that together bring together Rag GTPases *Raga* and *Ragb* with a subgroup of mTORC1 proteins termed the Regulator. Overall, this active state of mTORC1 inhibits the autophagy function of lysosomes that deliver cholesterol, long chain fatty acids and amino acids. The selection of cholesterol as a key mediator recognizes the importance of this multistep *Srebp2*-mediated synthesis as a consumer of ATP. Hepatocyte cholesterol is critical to cell signaling as a determinant of membrane fluidity.

The mTOR kinase which is central to this complex has multiple functions including the kinase activation of *Srebp* forms. However, recruitment of the complex to lysosome vesicles is not the key control point for the mTOR kinase activity. Rapamycin the specific inhibitor of the mTORC1 kinase activity prevents activation of *Srebp*-controlled genes but protein components of the mTORC1 complex determine the impact of the Slc38a9 complex on *Srebp* activities. Critically, mTORC1 kinase effects *Srebp* activations through an intermediate target S6 kinase. On the LF12 breeder diet the near complete dependence of these activities on *Cyp1b1* is remarkable. A second TOR1 complex (mTORC2) that regulates the effects of Akt activity is inhibited by S6 Kinase. This division of S6 Kinase effects may explain how *Cyp1b1* effect on *Srebp* appears in reverse on *H19* and *S100a9* which are promoted by a slowing of neonatal differentiation.

*Scd1* provides the best correlations between Srebp activities and growth on both BD and LF12 diets (Figure 6). *Hmgcr* has the weakest match, probably due to the *Insig*/*Scap* feedback suppression (Table 5 and Supplementary Tables). *Scd1* expression produces larger increments in the growth on BD for each unit of expression change compared to the LF12 diets. This extra growth promotion provided by BD factors must derive from different impacts of mTORC components [90]. Some of these factors emphasize differentiation more than growth [82]. Low mitochondrial generation of ATP is connected to the mTORC1 complex through enhanced AMP kinase activity. This key kinase inhibits mTORC components thus promoting nutrient availability through activation of lysosomal autophagy. Iron, ROS and NADPH link to redox effects on this ATP/AMP ratio (Figure 9).

Individual litters on the BD diet also show a growth-Srebp activity correlation but substantially displaced from the LF12 plot. Supplementation of LF12 with ALA to match the BD content shifted the correlation of growth and Srebp activity to the BD level (Table 6; Figure 7). Together this data suggests that these diets and particularly ALA affect the impact of mTORC1 on Srebp activation and growth outcomes.

Direct *Srebp* binding to the respective promoters has been established for the pathway genes. However, a shared functional mediator such as *mTORC1* may achieve a similar result without this direct *Srebp* interaction. TORC1 enhances cholesterol synthesis via enhanced binding of *Fam120A* to *Srebp* forms [91]. *mTORC1* also connects to ferroptosis via *Spebp1* control of *Scd1* [92] and to *Gpx4*, a ferroptosis control protein that is highly stimulated at birth in *Cyp1b1*^−/−^ pups [93].

### 3.7. Cyp1b1, Hepcidin (HepC), and Iron Regulation

The most interesting impact of *Srebp1*/*Cyp1b1*/VAD is the suppression of *Hamp*/Hepc, the prime regulator of iron distribution. This response involves near-total suppression of this iron regulator at birth, which is sustained at P21, even as locus co-regulation of *Hamp1* and *Hamp2* changes selectivity (Figure 2D). *Hamp1* is exclusively expressed at birth; there is a switch to *Hamp2* preference at P21, and then another switch to almost equal regulation in the adult male. From birth to P21, the joint regulation is subject to equal suppression by *Cyp1b1* deletion and WT/VAD treatment. The *Hamp* locus is regulated by *Srebp1* [33] in addition to *BMP6*/*Smad1* [30]. Importantly, the reversal of suppression in *Cyp1b1*^−/−^ pups by VAD treatment of the mother provides a significant reversal at birth that matches the dual HSC activation and the subsequent *Srebp* suppressions. Critically, while there are many exclusive *Cyp1b1* responses, essentially all VAD effects involve either selective parallel effects (*Srebp1* and HSC activation) or VAD reversal of *Cyp1b1*^−/−^ effects (all VAD affected genes).

Low *Hamp* expression is often taken as a measure of reduced availability of serum iron [28]. This is consistent with the lower *Hamp* with the base LF12 diet, which has lower iron than the BD diet. However, manipulations of the diet or *Cyp1b1* genotype may uncouple the *Hamp* expression process from the levels available during the EC/BMP6 sensing process. These putative stellate and *Hepc* changes are matched by a dominant advance in *Cyp1b1*^−/−^ mice in the expression of over 100 genes that appear later in the neonatal liver program. VAD has no effect on these responses.

Many of these genes show links to iron regulation, including ferritin light-chain subunits (*Ftl*1 and 2) that regulate ferroptosis and store iron [50,51], and Transferrin/*Trf*, which distributes iron into the hepatocyte. Glutathione peroxidase 4 (*Gpx4*), the prime marker of ferroptosis, is also highly elevated, although, like HSC activation, this extra increase is limited to the early neonatal period. Some genes that are suppressed follow that same timing advance. The growth factor granulin, which is very highly expressed at birth, is completely removed in *Cyp1b1*^−/−^ liver at birth, but also declines extensively in the developing liver.

### 3.8. Cyp Genes Play a Unique Role in Sustaining Neonatal Female Polarization of Neonatal Liver

About 40 percent of 102 *Cyp* genes in the mouse genome are appreciably expressed in neonatal mouse liver. An unbiased microarray strategy identified 200 genes exhibiting male/female expression bias (M-DIM or F-DIM; adult expression ratio > 2.5 ). This sexual expression bias which develops in males after puberty, included 18 *Cyps*. M-DIM genes increase while F-DIM genes decline. There is a strong preference for F-DIM selectivity among these *Cyps* (14/4). Remarkably, most of the F-DIM *Cyps* (11/14) were suppressed in *Cyp1b1*^−/−^ livers at 14 week while the responsive M-DIM males showed stimulation (2/4). These *Cyp1b1* effects were not evident prior to age 8 week [43]. Thus, these DIM changes are age dependent through *Cyp1b1*-dependent processes. Genes that show stable DIM (*Cyp2c* forms also lack *Cyp1b1* sensitivity. This DIM instability is seen with the BD diet to a greater extent than with those bred on the LF12 diet. Thus, ALA which is key to the distinction between BD and LF12 diets may continue to impact the adult development even after maturity. Preliminary data suggests that pre-pubertal removal of ALA has similar effects on adult liver changes as *Cyp1b1* deletion.

In the adult, the M-DIM and F-DIM associations also showed a close correlation between deletion effects of *Hnf4a* and *Cyp1b1* [42]. This selectivity confirms the findings of previous pioneering studies [43]. The respective deletions produce highly correlated adult liver expression changes. *Hnf4a* is a marker of the developing sinusoids [24,27]. Among these dimorphic genes, only the F-DIM genes in neonatal liver included 13 *Cyps*. This F-DIM bias is amplified by their linkage to specific lipid-activated receptors (PPARα, CAR, and PXR), which control many other DIM genes [76].

EET oxylipins can often function as ligands for these nuclear receptors. Their activities are often transient and therefore highly localized, even with cells due to an abundance of soluble epoxide hydrolase [47,75]. ALA is a critical dietary source for such ligands that cannot be generated from the *Srebp1*-regulated pathway. *Cyp1b1* shares this lability for may be functionally important for oxylipin mediation of inflammatory (monocyte), vascular (pericytes/HSCs/ECs), or neuronal (microglia/astrocytes) effects. Such considerations are frequent for local vascular oxygen fluxes where Cyp1b1 effects have been established [6,21,22].

### 3.9. Defined Diets Identify a Novel Cooperation Between Cyp1b1, ALA, and Iron Homeostasis

The defined series of LF12 diets with and without ALA and iron have established extensive coupling between Cyp1b1, ALA, and iron. Dietary ALA is converted to both n-3 C20 eicosapentenoic acid (EPA, n-5) and C22 n-3 docosahexaenoid acid (DHA, n-6) occurs through the *Elovl* forms (3 and 5) and *Fads* desaturases (1 and 2) (Figure 8). Metabolism of all three ω3-fatty acids by *Cyp1b1* generates oxylipins that can activate lipogenesis through PPARγ [35] or other receptors and channels [47,75]. *Cyp1b1* in a subset of activated HSCs generates partners with *Scd2* to generate a specific activator of the Leukotriene B receptor (*Ltb4r2*). This receptor activation promotes liver tumor growth [46]. The TRPV1 Ca-channel (Capsaicin-activated pain mediators) is commonly associated with ALA-linked oxylipins. Mediation by *Cyp1b1* has been identified in human pain responses [78,87]. This channel is highly expressed in endothelia and this route has been linked to *Hamp* stimulation [94]. Interestingly, C18 elongation enzymes *Elovl5* and *Fads1*, which convert C18 fatty acids to C20 fatty acids, are strongly expressed. *Fads1* is among the genes in the *Ftl1*-*Trf* cluster and is enhanced 5-fold in *Cyp1b1*^−/−^ pups at birth.

The use of a defined LF12 diet established the dominant overlapping suppression of *Srebp1* signaling by *Cyp1b1*^−/−^ and VAD. Supplementation by iron or ALA to BD levels established that each of these can reverse these effects, although the specific processes involved depend on the individual C57Bl/6j mother. The contrast between the similarity of littermates and the diversity of the mothers’ points to strong epigenetic intervention of the type provided by *LncRNA H19*.

Individual offspring from 40 litters comprising eight treatment groups (WT and *Cyp1b1*^−/−^ mothers with four diets) were separated into three growth populations with diverse representation from the eight treatment groups. BD litters were more prevalent in the high BW group, whereas *Cyp1b1*^−/−^ litters were more represented in the low BW group. LF12 diets with iron or ALA supplementation were better represented in the high-growth group but also generated litters with full separation of BW. We termed these growth divisions enhanced, medium, and survival (EG, MG, and SG). The survival term is used because even low-growth representatives of the SG group recovered the mean range of BW at 14 weeks of age. Most treatments resulted in a dominant clustering with outliers.

The mothers fed the LF12 diet also produced a greater diversity of sizes at 9.5 days post conception, which marks the start of liver development, despite indistinguishable morphology markers (somite counts) [8]. The exceptional proportion of smaller E 9.5 embryos born to mothers fed the LF12 diet indicates that this diet led to slower growth.

Neonatal liver development is supported by diverse cell types external to the hepatocytes including the initial LSEC vasculature. Remarkably, *Cyp1b1* plays a modulating role in each of the five sites, including the liver-initiating STM (Figure 9). We have not addressed the sympathetic nervous system here, but previously showed that *Cyp1b1* controlled estrogen-linked mediation of leptin activity that was directed by transfer across the blood–brain barrier to the Ventral Medial Hypothalamus [42]. A local endothelial increase in Bmp6 to direct estrogen promoted *Hamp*/HepC expression [30]. We have provided evidence of the intervention of specialized granulocytic monocytes in lactation driven by the Ca-binding proteins S100a8 and S100a9 [83].

This model identifies a set of markers for multiple sites of *Cyp1b1* intervention in neonatal liver development. We have introduced some new approaches by emphasizing the importance of using suboptimal diets and documenting individual litters. We recognize the need to use as many as ten litters for each treatment. Figure 9 documents multiple, mechanistically different markers. Multiple sites of expression are implicated in ECs, HSCs, and monocytes. The changes are local and dynamic. *H19* and mediating miRNAs that target both provide restraints to define both location and timing.

## 4. Materials and Methods

### 4.1. Animals

Colonies of wild-type and *Cyp1b1*^−/−^ mice derived from C57BL/6J mice (Jackson Laboratory, Bar Harbor, ME, USA) were maintained in the AAALAC-accredited laboratory of the University of Wisconsin Medical School, according to ACUC protocol number M005635. *Cyp1b1*^−/−^ mice were previously backcrossed through 5 generations with WT mice (N5 *Cyp1b1*^−/−^ mice). For defined breeding, WT or N5 nulliparous female mice (aged 8–12 weeks) were time-mated with matched males to obtain a vaginal plug at the time designated E0.5. Throughout this breeding and up to E4.5, the pregnant females were fed standard breeder diet BD2019 (Harlan, Indianapolis, IN, USA). Litters were maintained on BD. At weaning (P21), pups were removed from the mother and maintained a low-fat (10%), high-carbohydrate (68%) diet (LFD) provided by Research Diets (product number 12450B). The mice were euthanized individually with CO_2_ at birth, weaning week 8, or week 14, as specified. At birth, tissues from the livers of the whole litter were combined. Body and liver weights were determined for individual mice and were matched to gene expression PCR data.

### 4.2. Defined Diets

At E4.5, the mother and litters were transitioned to defined LF12 diets (vitamin A sufficient (VAS) (Harlan, product number 07655). BD had soybean oil (SBO; 22%) as its fat source, whereas for LF12, the fat source was cottonseed oil (CSO; 12%). VAS BD contained vitamin A/retinol at 24,000 IU/kg of retinyl palmitate, whereas the vitamin A-deficient (VAD) diet had 100 times less (220 IU/kg). Retinol and retinol ester levels were determined for all liver samples and are presented in the work of Maguire et al. [34]. A major difference between BD and LF12 derived from the fat source. CSO in the LF12 diet is deficient in α-linolenic acid (ALA), the major ω3-fatty acid. LF12 also contains the iron content of LFD and therefore lacks the 3-fold iron supplement that is applied to BD2019. To match the iron content of BD in the LF12 diet, the iron content was increased by 3-fold (LF12-Fe) (Teklad, product number 160682). To match the ALA content of BD, the CSO was supplemented with flaxseed oil, which was composed of 55% ALA but otherwise had similar proportions of fatty acid esters to SBO (Table S5).

### 4.3. RNA Isolation

The frozen liver thawed in RNAlater Ice (ThermoFisher, Carlsbad, CA, USA). Total RNA was isolated from 20 mg of tissue using an RNeasy Mini kit, followed by elution from Qiashredder columns (Qiagen, Valencia, CA, USA). RNA purity was assessed based on the A280/A260 ratio on a Nanodrop.

### 4.4. Microarray mRNA Profiling

Microarray analyses were carried out on two pooled litters at birth for each treatment group. At weaning (P21), RNA was analyzed from three individual mice in each treatment group. RNA was analyzed from livers of WT and *Cyp1b1*^−/−^ mice, each maintained on LF12, both VAS and VAD. The *Cyp1b1*^−/−^ mice were from the N5 colony. Samples were prepared for single-color Cy3 labeling and applied to an Agilent Technologies 4x44 platform (Madison, WI, USA). The data were deposited in the NCBI gene Expression Omnibus (Accession number GSE87844). The data are presented in the following ratios: *Cyp1b1*^−/−^/WT (each VAS); WT VAD/VAS; *Cyp1b1*^−/−^ VAD/VAS. For each gene, fold change (FC) and *p*-value determinations were made using LIMMA Statistics. FC (ln_2_) is plotted versus *p*-values (log_10_) in Volcano Plots (Figure 2A).

### 4.5. Systematic Color Coding in Tables

The data derived from Wild type and *Cyp1b1*^−/−^ mice together with several different diets is complex but the same groupings are repeated in multiple Tables. We have repeated color coding across different Tables and Figures to aid in the recognition of response patterns.

Table 1, Table 2, Table 3 and Table 4 each derive from micro-array data carried out in triplicate. Effects of *Cyp1b1^−/−^* versus VAD at birth and weaning (P21). P21 is seven days prior to puberty (P28). All effects of switch from WT to *Cyp1b1*^−/−^ are coded in **yellow**. Effects of VAD on WT mice from E4.5 are coded in **green**. Effects of VAD on *Cyp1b1^−/−^* mice are coded in **blue**. Statistics for these changes are derived from full array LIMMA readouts; ** *p* < 0.01 and * *p* < 0.05. All fold changes have *p* < 0.01 negative FC indicating fold decrease are italicized. Genes highlighted in** red** are those further addressed in Figures and Text.

Table 5 and Table 6 use RT PCR (ΔCt relative to ΔCt for β-Actin) compare select markers for Srebp1 and Srebp2 activities to those of Hamp forms which encode pro-Hepcidin. Table 1, Table 2, Table 3 and Table 4 were each carried out with LF12 breeder diet while Table 5 and Table 6 use breeder diet BD2019, LF12 diet or Supplemented LF12 as specified. Thus, array data can be directly compared to PCR data carried out with LF12 diet.

Table 7, Table 8 and Table 9 examine multiple Cyps using the same birth and weaning arrays as were used for Table 1, Table 2, Table 3 and Table 4. Adult expressions were derived from separate breeding using BD 2019 and adult defined LFD. We focus on how dimorphic expression (DIM) in the adults determined at age 8 week in males and 14 week in females. *CYPs* showing expression bias in females (F-DIM) are color coded in **green** highlighting expression at birth, P21, and week 14. *Cyps* showing expression bias for males (M-DIM) are color coded in **red**.

### 4.6. Quantitative Real-Time PCR (qRT-PCR) Analyses

qRT-PCR analyses were carried out on RNA from liver samples from individual mice in litters at weaning (P21). RNA was isolated from BM-MSC by the standard Trizol (ThermoFisher) and Qiagen RNeasy Mini kit procedures (Qiagen). cDNAs were generated using random hexamers and GoTaq polymerase (Promega, Madison, WI, USA), according to the manufacturer’s protocol. The expression levels of *9* genes were determined by qPCR using the primer pairs shown below. For each gene, an ΔCt shift relative to β-actin was determined. The primers used were as follows: *Actb2* (β-actin) Forward: 5′-CAACGAGCGGTTCCGATG-3′ and Reverse: 5′-GCCACAGGA TTCCATACCCA-3′; Acss2 Forward: 5′-ACCAGTTAAGAGGCCATGTC-3′ and Reverse: 5′-GTACAAGATGAAGAGTGGGTC-3′; *Elovl6* Forward: 5′-GAACAAGCGAGCCAAGTTTG-3′ and Reverse: 5′-TGTAAGCACCAGTTCGAAGAG-3′; *Fasn* Forward: 5′-GCTGCGGAAACTTCAGGAAAT-3′ and Reverse: 5′-AGAGACGTGTCACTCCTGGACTT-3′; *Hamp*/Hepcidin (same forward primer for both reverse primers) Forward: 5′-CTGAGCAGCACC ACCTATCTC-3′, Hamp1 Reverse: 5′-TGGCTCTAGGCTATGTTTTGC-3′, and Hamp2 Reverse: 5′-GGCTCTAGGCTCTCTATTCTTCA-3′; *Hmgcr* Forward: 5′-GCCCTCAGTTC AAATTCACAG-3′ and Reverse: 5′-TTCCACAAGAGCGTCAAGAG-3′; *Me1* Forward: 5′-AGTATCCATGACAAAGGGCAC-3′ and Reverse: 5′-ATCCCATTACAGCC AAGGTC-3′; *Scd1* Forward: 5′-TTCTTGCGATACACTCTGGTGC-3′ and Reverse: 5′-CGGGATTG AATGTTCTTGTCGT-3′; *Sqle* Forward: 5′-CCCCAAAACACAAAATCCT CAG-3′ and Reverse: 5′-GCAATGCCAAGAAAAGTCCAC-3′.

### 4.7. Statistics

#### 4.7.1. Application of Linear Model for Microarray and RNA-Seq Data (LIMMA) Analyses for Large Gene Number/Small Repeat Data Sets

Statistical evaluation of the effects of different treatment groups at each array site was performed using the EDGE platform, which employs several built-in algorithms that are integrated with Bioconductor, an R-based open-source statistical genomics collaboration. This approach focuses on the LIMMA processing of a full matrix of expression values. Each row represents a gene; each column corresponds to an RNA expression level (Cy3 binding) derived from a treatment group. Comparisons are made between treatment groups represented as fold change and *p*-values, which are also presented in Volcano Plots (Figure 1). This approach leverages the highly parallel nature of genomic data to estimate different levels of variability between genes and samples. This extension improves the reliability when treatment sample numbers are small and effectively clusters gene expression signatures.

The LIMMA approach analyzes experiments in their entirety rather than being limited to pairwise treatment comparisons. The Empirical Bayes method borrows information delivered by trends within the whole 45,000 microarray set. Thus, the global variance allows for increases in variance when the expression is lower. LIMMA also includes a background correction function that improves reads at low levels of expression that dominate these measurements. The LIMMA approach has appreciably expanded in currently available formulations [95,96].

#### 4.7.2. Statistical Analysis of qRT-PCR Data

Statistical significance was determined by ANOVA with Tukey’s post hoc test for multiple comparisons, *p* < 0.05; trending was defined as 0.05 < *p* < 0.1 (GraphPad Prism 10, San Diego, CA, USA).

## Figures and Tables

**Table 1:** Expression changes seen in *Cyp1b1^−/−^* pups at birth (P1).

Gene	Exp P21 Exp × 10^3^	Exp P1 Exp × 10^3^	FC P21/P1	*1b1* ^−/−^ / WT P1	WTVAD WT P1
**Stellate Activation**					
***Col1a1***	**1.3**	**0.9**	**1.4**	4.5 *	4.1 **
***Col1a2***	7.0	4.7	1.5	4.6 **	2.6 *
***Col3a1***	4.2	2.9	1.5	2.5 ^#^	2.8 ^#^
***Acta2***	**1.0**	**0.35**	**3**	2.6 *	**2.0 ^#^**
***Fbln2***	0.4	0.5	0.8	2.1	2.4
***Cyp2s1***	0.3	0.15	2	1.5	2.6 **
***Timp2***	2.9	2.3	1.3	1.9 *	1.7 ^#^
***Tgfb3***	0.3	0.2	1.5	1.6	1.8 ^#^
**Iron/Ferroptosis**					
***Hamp/Hamp2 ^a^***	23 ^#^	31	−1.3	−8.8 **	−2.5 *
***Slc40a1/Fpn***	4.1	10.5	−2.5	−1.7 *	nc
***Hmox1***	1.2	2.2	−1.9	3.4 **	nc
***Ftl1***	**23**	**2.7**	**9**	9.7 **	**nc**
***Ftl2***	42	5.5	8	9.7 **	nc
***Trf***	**123**	**37**	**4**	5.1 **	**nc**
***Tfrc***	0.36	0.8	2.2	nc	nc
***Hfe2***	**0.3**	**0.05**	**6**	4.0 **	**nc**
***Gpx4***	**13.7**	**11.4**	**nc**	3.0 **	**nc**
**Perinatal *1b1*^−/−^ Transition **					
**Suppression**					
***Granulin/Grn***	17	190	−11	−150 **	nc
***Syt10***	0.2	2.2	−11	−60 **	nc
***Klf2***	26	59	−2.3	−4.8 **	nc
***Dusp8***	33	66	−2.0	−4.8 **	nc
**Stimulation**					
***Aldh2***	14	0.4	40	17.5 **	nc
***Serpina1b***	48	5.3	9	11.8 **	nc
***Cyp2d12***	14	2.1	6.5	11.5 **	nc
***Atp5b***	6.7	1.1	6	9.9 **	nc
***Khk***	19	0.3	55	6.6 **	nc
***Fads1***	4.5	0.6	7.5	5.3 **	nc
***Aldob***	44	11	4	4.5 **	nc
***Eif5a***	5.8	2.3	2.5	6.0 **	nc

Expression changes were of three types: (A) Stellate Activation, (B) Iron regulatory changes, and (C) Perinatal Advancement. Vitamin A deficiency (VAD) matched *Cyp1b1* deletion in stellate activation. The Perinatal Advancement responses to *Cyp1b1* deletion (*1b1*^−/−^/WT) match the advancement seen between birth (P1) and weaning (P21; FC P21/P1) (see Figure 1). ^a^ Sum of Hamp1 and Hamp2 (stimulation and suppression). Genes highlighted in **red** are those further addressed in Figures and Text. FC: fold change, nc = no change. ^#^ *p* = 0.1; * *p* < 0.05; ** *p* < 0.01.

**Table 2:** Effects of *Cyp1b1* deletion at Birth on genes that affect *H19* expression or function.

	P21	P1	P21/ P1	*1b1*^−/−^/ WT	WT VAD WT
	**Exp × 10^3^**	**Exp × 10^3^**	**FC**	**P1**	**P21**
					
***H19***	**4.2**	**74**	−18 **	1.7 *	**nc**
					
***Cnot3***	**1.4**	**2.1**	**−1.5**	−1.75 *	**nc**
*Ago2*	1.0	4.1	−4	−1.6 ^#^	nc
*Pan3*	0.8	1.4	−1.8	−2.2 **	nc
					
*Cdk4*	0.4	0.3	nc	4.6 **	nc
***Ezh2***	**0.08**	**0.14**	**nc**	2.35 *	**nc**
*Zhx3*	3.4	2.6	nc	−1.8 ^#^	nc
*Ap2m1*	1.2	0.7	1.7	2.3 **	nc
					
***H1fx***	**9**	**18**	**−2**	−3.2 **	**nc**
*Hist1h1b*	0.7	7.3	−10	4.1 **	nc
*Hist1h1c*	1.5	5.8	−4	−3.3 **	nc
*Hist1h2ak*	1.8	4.4	−2.5	6.8 **	nc
*Hist3h2a*	1.5	8.2	−5.5	3.5 **	nc

*H19* modulators are divided into three groups: mRNA suppressors; histone3 methylation modulators and Histone1 modifiers. ^#^ *p* = 0.1; * *p* < 0.05; ** *p* < 0.01 (stimulation and suppression). FC: fold change; nc: no change. Genes highlighted in **red** are those further addressed in Figures and Text.

**Table 3:** Stimulations at weaning/P21 seen in *Cyp1b1^−/−^*mice. Reversal when VAD is combined with *Cyp1b1^−/−^*.

Gene	Exp P21 ×10^3^	Exp P1 ×10^3^	FC P21/P1	*1b1* ^−/−^ / WT P21	WT VAD WT P21	*1b1*^−/−^ VAD *1b1*^−/−^ P21
*Lyz2*	7.8	42	−5.5 **	nc	nc	nc
*Tfrc*	0.36	0.8	−2.2 **	2.2 **	nc	−1.55 *
						
***H19***	**4.2**	**74**	−18 **	8 **	**nc**	−4 *
*Igf2*	0.07	108	−1400 **	30 **	nc	−14 **
*Afp*	0.5	276	−550 **	18 **	nc	−4 **
						
***S100a9***	**1.25**	**207**	−170 **	4 **	**nc**	−4 **
*S100a8*	0.2	42	−200 **	3.4 **	nc	−4 *
						
*Igfbp2*	21	90	−4.5 **	3 *	nc	−1.3
*Krt23*	1.1	6.0	−5.7 **	3 **	nc	−2
						
***Hist1h1b***	**0.7**	**7.1**	−10.2 **	2.9 **	**nc**	−2.0 **
*Hist1h2ak*	1.8	4.4	−2.5 **	2.4 ^#^	nc	−1.7 *
*Hist1h2ai*	1.2	2.9	−2.5 **	2.7 ^#^	nc	−1.7 *
*Hist3h2a*	1.5	8.2	−5.5 **	2.0 **	nc	−1.8 **
						
*Cyp3a16*	3.1	7.1	−2.2 *	3 **	nc	Nc
*Gpc3*	0.65	6.9	−10	2.3 **	nc	−1.2

# *p* = 0.1; * *p* < 0.05; ** *p* < 0.01. FC: fold change; nc: no change. Genes highlighted in** red** are those further addressed in Figures and Text. Effects of *Cyp1b1^−/−^* vs. WT are coded in yellow. Effects of VAD on WT is in green. Effects of VAD on *Cyp1b1^−/−^* pups is in blue.

**Table 4:** Suppression effects of *Cyp1b1*^−/−^ and VAD on select activities controlled by *Srebp1* and *Srebp2*. Reversal when VAD is combined with *Cyp1b1^−/−^*.

Gene	Ratio P21/P1	*1b1*^−/−^ WT	WT VAD WT	*1b1*^−/−^VAD *1b1*^−/−^
**Fatty**	**Acids**			
***Srebp1c***	4.9 **	nc	−1.4	Nc
***Mlxipl***	3.3	−2.6 **	nc	Nc
***Acss2***	8.5 **	−8 **	−3.4 **	2.4 **
***Me1***	8.4 **	−5 **	−4 **	1.4 *
***Fasn***	28.3 **	−12 **	−3.3 **	2.6 **
***Scd1***	86.9 **	−12 **	−4.3 **	1.9
***Elovl6***	6.0 **	−28 **	−8 **	2.8 **
**Other**	**Srebp1c**	**Genes**		
***Hamp2***	187 **	−28	−50 **	3.7 **
***Rdh11***	3.0 **	−5.0 **	−2.3 **	3.3 **
***Mup3***	>100	−34 **	−5 **	3.5 **
***Gadd45g***	6.2 **	−6.6 **	−6.7 **	2 **
***Lpin1***	3.6 **	−4.5 **	−2.9 **	Nc
***Rgs16***	2.2 **	−5.9 **	−3.0 **	1.6 **
**Cholesterol**	**Pathway**			
***Srebp2***	nc	nc	nc	Nc
***Sqle***	2.4	−24 **	−2.2 **	26 **
***Hmgcr***	4.0	−6.4 **	−1.4	4.2 **
***Insig1***	7.8	−3.55	nc	4.2 **
***Pcsk9***	4.2	−4.7 **	nc	6.6 **
***Stard4***	1.6	−2.3 **	nc	2.1 **

Srebp1 and Srebp2 activities increase from birth to weaning but are suppressed in *Cyp1b1^−/−^* mice (yellow). VAD directly suppresses the Srebp1c processes (green) but not Srebp2 processes. VAD treatment of *Cyp1b1^−/−^* decreases the suppressions (blue). * *p* < 0.05; ** *p* < 0.01. nc: no change. Genes highlighted in** red** are those further addressed in Figures and Text. All effects of switch from WT to *Cyp1b1^−/−^* are coded in **yellow**. Effects of VAD on WT mice from E4.5 are coded in **green**. Effects of VAD on *Cyp1b1^−/−^* mice are coded in **blue**.

**Table 6:** Iron and *Cyp1b1* shift LF12 growth outcomes without changing the pattern of *Srebp1* activities (see Figure 6). ALA switches outcomes typical of a BD response program.

	BW	Elov6	Scd1	Fasn	Hamp
**EG**					
**BD**	10.5	3.8	−0.9	1.5	−1.3
**BD-*1b1***	9.2	5.2	−0.7	2.6	0.7
**LF12-ALA**	**10.5**	**3.1**	**−0.8**	**3.3**	**1.5**
**Mean ΔCt**		*4.5*	*−0.8*	*2.5*	
					
**LF12-Fe**	**9.1**	**1.7**	**−4.6**	**−0.1**	**1.1**
					
**MG**					
**BD-*1b1***	7.8	7.0	2.0	3.2	1.2
					
**LF12**	**7.9**	**2.0**	**−0.8**	**−0.8**	**1.2**
**LF12-Fe**	**8.0**	**1.2**	**−2.9**	**1.3**	**1.1**
**LF12-Fe** **-*1b1***	**8.4**	**3.1**	**−1.4**	**1.8**	**−1.4**
**Mean ΔCt**		*2.1*	*−1.7*	*0.8*	
					
**SG**					
**BD-*1b1***	6.8	7.0	1.8	3.7	−1.3
**LF12 ALA**	**6.3**	**6.9**	**4.5**	**5.6**	**1.0**
					
**LF12**	**6.0**	**4.5**	**2.4**	**4.0**	**5.2**
**LF12-*1b1***	**6.4**	**6.6**	**3.7**	**3.2**	**8.0**
**LF12-Fe**	**6.4**	**5.2**	**2.4**	**3.8**	**3.3**
**LF12-Fe** **-*1b1***	6.6	5.8	1.8	3.7	2.4
**Mean ΔCt**		*5.5*	*2.6*	*3.7*	

For Srebp1 markers and EG group LF12-ALA expression matches expression of BD litters rather than expression of LF12 or LF12-Fe. For SG group there is no diet effect (BD or LF12) for Srebp markers or BW. For Hamp retains high expression for LF12-ALA and BD despite low Srebp values. The shared ALA component effects uncoupling of Hamp from Srebp activity (see Bmp6 route Hamp activation). The similarity of Srebp activities irrespective of treatment is consistent with the SG group as a program in which dietary resources are focused on developmental survival.

**Table 7:** Multiple P450 Cytochromes that show adult F-DIM and M-DIM polarizations are expressed in neonatal liver.

*Cyp*	Birth	wk3	wk14	*CypP*	Birth	wk3	wk14
	Cy3 × 10^3^	Cy3 × 10^3^	*1b1^−/−^ WT* (FC ^#^)		Cy3 × 10^3^	Cy3 × 10^3^	*1b1^−/−^ WT* (FC ^#^)
**DIM Neutral**						**F-DIM**	
***2a12***	13	23	nc	**2a4**	13	38	−4.6 *
***2a22***	1.1	3.5	nc	**2a5**	14	39	−4.4 **
***2c29***	16	33	1.5	**2b9**	5.0	22	−25 HF ^#^
***2c37***	7.5	15	nc	**2b13**	0.6	4	−60 HF ^#^
***2c40***	54	5	nc				
***2c44***	8.5	4	nc	**3a11**	3.0	7	−3.7 *
***2c70***	14	53	2.5 **	**3a16**	7.0	3	−5.6 **
				**3a41**	**57**	**50**	−5 **
***3a13***	11	5	nc	**3a44**	6.0	5	−4 **
***3a25***	1.0	9	nc				
				**4a10**	66	16	−9 **
***4f13***	0.2	1	nc	**4a14**	155	40	−64 **
***4f14***	0.1	13	nc				
***4f15***	3.4	5	nc	**39a1**	7	4	−2.9 **
***4f18***	0.2	0.7	nc				
***4v3***	1.0	3.6	**2.5**			**M-DIM**	
				**4a12a**	0.1	0.02	**Nc**
				**4a12b**	0.6	0.7	**Nc**
***2d10***	133	102	nc	**7B1**	0.1	0.5	6 **
***2d12***	2	14	nc	**27a1**	0.02	0.9	2.1(HF) *
***2d13***	0.2	1.5	nc	**2U1**	0.05	0.2	**2.5**
***2d22***	0.2	2.5	1.5				
***2d26***	249	173	nc			**Other**	
***2d34***	1.4	3	nc	**7a1**	0.4	0.8	−2.5
***2d9***	67	68	nc	**8b1**	0.04	0.3	Nc
				**17A1**	3.5	3.5	Nc
***2e1***	11	39	1.5	**26A1**	nc	nc	Nc
***2f2***	22	36	1.5	**26B1**	0.4	0.8	4
							
***2j5***	2	6.5	nc	**1B1**	<0.02	0.05	Nc
							
*1a1*	<0.1	<0.1	**nc**				
*1a2*	0.04	1.5	1.5				

Neonatal expression (Birth-Weaning/p21) is compared for neutral (Left side) and Dimorphic Cyps (Right side). F-DIM and M-DIM polarizations are linked to ***Cyp1b1*^−/−^/WT** expression ratios. **HF** response only seen on the high fat diet which greatly increased expression. # *p* = 0.1; * *p* < 0.05; ** *p* < 0.01.

**Table 8:** Dimorphism of P450 Cytochromes based on adult male and female expression matches effect of *Cyp1b1*^−/−^ at 14wk in males.

Ages 8w and 14 w	Expression 8w-M	DIM 8w-M/14w-F	KO-LFD 14w	Weaning	Birth
**Genes**					
***1b1***	**responsive**				
***Cyp2a4***	6.4	−6.9	−4.0	38	14
***Cyp2a5***	10.3	−4.5	−4.0	39	13
***Cyp2b13***	0.01	−440	−25(H)	4.0	0.6
***Cyp2b9***	0.002	−3780	−60(H)	22	5.0
***Cyp39a1***	0.35	−2.9	−3.5	3.7	7.0
***Cyp3a11***	2.0	−5.9	−2.0	7.4	2.8
***Cyp3a16***	**0.5**	**−23.6**	−2.5	**3.2**	**7.1**
*Cyp3a41*	**15.0**	**−7.4**	−2.5	**50**	**57**
***Cyp3a44***	**1.1**	**−8.2**	−2.0	**5.5**	**6.4**
***Cyp4a10***	**1.3**	**−7.3**	−9.0	**16**	**66**
***Cyp4a14***	**0.17**	**−110**	−50	**40**	**155**
***Cyp7b1***	**18.4**	**12.4**	**9.0**	**0.45**	**0.08**
***Cyp2u1***	**0.9**	**5.2**	**2.5**	**0.2**	**0.05**
					
***1b1***	Independent				
***Cyp2c29***	11.4	−2.7	nc	33	16
***Cyp2c37***	2.2	−3.9	nc	15.4	7.4
***Cyp2c40***	14	−3.8	**nc**	58.0	54.0
***Cyp4a12a***	**7.4**	**258**	**nc**	**0.02**	**0.1**
***Cyp4a12b***	**2.6**	**6.9**	**nc**	**0.6**	**0.7**

Expression changes from weaning to maturity match to *Cyp1b1*^−/−^/WT ratios. F-DIM genes decrease expression from weaning (3 weeks) to maturity (8 weeks). For many F-DIM genes, expression is sustained at birth. M-DIM genes increase expression from weaning to maturity but lose neonatal expression. DIM Cyps all show matching DIM and *Cyp1b1*^−/−^/WT ratios.

**Table 9:** Male neonatal expression is dominated by F-DIM expression.

Age/Sex Gene. Cy3 × 10^3^	Unresolved Birth	Male Weaning	Optimum Male 8 wk	Constant Female 14 wk
**M-DIM**				
***1b1* -positive**				
***Moxd1***	<0.01	<0.01	**1.8**	<0.02
***Cyp7b1***	0.1	0.5	**18**	2
***Hsd3b5***	0.4	0.3	**14**	0.06
***Hsd3b4***	0.1	0.25	**17**	0.1
***Ces2b***	0.45	0.2	**2.0**	0.5
***Slco1a1***	<0.01	<0.01	**0.7**	0.025
				
***1b1*** **-insensitive**				
***Cyp4a12b***	**0.7**	0.6	**2.6**	0.4
***Elovl3***	**0.01**	0.02	**6.6**	0.02
				
***F-DIM***				
***1b1* -negative**				
***Ly6c2***	7.0	7.0	1.4	10
***Acot4***	1.0	0.35	0.06	0.5
***Cyp3a16***	7.0	3.2	0.5	12
***Cyp3a41***	57	50	15	109
***Cyp4a10***	66	16	1.5	15
***Cyp4a14***	155	40	0.15	30
***Cyp2a4***	14	38	6.4	42
***Cyp2a5***	13	39	10.3	45
				
***1b1*-insensitive**				
***Sult2a6***	12	30	<0.01	14
***Fmo3***	<0.01	13	<0.01	5
***Cyp2c37***	7.4	15	2.2	8
***Xist (8055)***	2.9	0.1	0.002	1.0
***Cux2***	0.7	0.3	0.003	0.2

In males, M-DIM genes are high at 8 weeks but low at both birth and weaning. F-DIM genes are high at weaning (P21) and at birth. This pre-pubertal expression parallels that in mature females.

## Data Availability

All the data are presented in the manuscript and Supplementary Data.

## Supplementary Materials

The following supporting information can be downloaded at https://www.mdpi.com/article/10.3390/ijms26052011/s1.
