# Supplementary material for: Defined Diets Link Iron and α-Linolenic Acid to Cyp1b1 Regulation of Neonatal Liver Development Through Srebp Forms and LncRNA H19
Source: Int J Mol Sci. 2025 Feb 25;26(5):2011. doi: 10.3390/ijms26052011 (PMC11901102; doi:10.3390/ijms26052011)
Supplement: Supplementary file 1 [file ijms-26-02011-s001.zip › Supplemental Tables S1-S5_1.20.pdf]

Supplemental Tables S1–S5

Table S1. SREBP1c regulated fatty acid synthesis genes compared to Srebp2 regulation of cholesterol synthesis genes. Each show reversal of Cyp1b1 deletion by co-treatment with VAD. VAS is normal retinol

Gene	Cy3 wk3	Cy3 Birth(B)	Ratio wk3/B	1B1-/- WT	VAD WT	VAD/ 1B1-/-
**Fatty**	**Acids**					
Srebp1c	34061	8766	4.9**	nc	-1.4	nc
Acss2	3220	364	8.5**	-8**	-3.4**	2.4**
Acly	6944	731	10.8**	-8.5**	-2.6**	1.5
Aacs	4385	509	10.4**	-17**	-2.7**	4.5**
Acacb	1911	268	7.9**	-12**	-3**	1.7
Acaca	1237	99	7.6**	-3.5**	-2.4**	1.6*
Me1	1760	324	8.4**	-5**	-4**	1.4*
Fasn	132152	6563	28.3**	-12**	-3.3**	2.6**
Scd1	18785	153	86.9**	-12**	-4.3**	1.9
Elovl6	7676	1499	6.0**	-28**	-8**	2.8**

**Other**	**Genes**					
Hamp1	**4493**	30996	5.9**	-78**	-79**	9.6**
Hamp2	**18303**	51	187**	-28	-50**	3.7**
Rdh11	**3146**	1068	3.0**	-5.0**	-2.3**	3.3**
Adh4	**4078**	227	20**	-1.8*	nc	1.5*
Mup1	**2595**	<50	>50	-4.1**	-3.1**	2**
Mup3	**16360**	<50	>100	-34**	-5**	3.5**
Mup4	**3326**	<50	>100	-7.3**	-4**	2**
Mup5	**983**	<50	>20	-6.2**	-4.5**	3**
Gadd45g	**11356**	2175	6.2**	-6.6**	-6.7**	2**
Lpin1	**3066**	997	3.6**	-4.5**	-2.9**	Nc
Rgs16	**3218**	1732	2.2**	-5.9**	-3.0**	1.6**
Hnf6	**3390**	2050	1.6	-1.9**	-1.4	1.6*

Gene	Exp wk3	Exp Birth(B)	Ratio wk3/B	1B1-/- WT	VAD WT	VAD/ 1B1-/-
Srebp2	4534	3528	nc	nc	nc	Nc
Sqle	2365	1434	2.4	-24**	-2.2**	26**
Idi1	5793	1974	4.7	-10.5**	-1.6	10**
Mvd	1287	259	4.3	-7.5**	-1.4	7.4**
Sc4mol	11976	5652	3.5	-7.1**	nc	7.4**
Fdps	4124	86	17.0	-6.4**	nc	8.7**
Dhcr7	10116	3461	3.7	-5.8**	-1.6	3.6**
Cyp51	550	110	3.6	-5.5**	-1.7	8.4**
Hmgcr	2919	835	4.0	-6.4**	-1.4	4.2**
Hmgcs	19776	8731	3.5	-4.7**	-1.4	3.8**
Lss	817	155	4.2	-3.9**	-1.8	5.1**
Insig1	1340	178	7.8	-3.55	nc	4.2**
Pcsk9	1159	159	4.2	-4.7**	nc	6.6**
Stard4	3937	2400	1.6	-2.3**	nc	2.1**
Ldlr	4045	1856	2.1	-1.7*	nc	2.0**

Table S2. Responses to resistant *Cyp1b1*$^{-/-}$ (R-*Cyp1b1*$^{-/-}$)

**Gene**	**Exp P21**	**Exp Birth(B)**	**Ratio# P21/Birth**	***Cyp1b1***$^{-/-}$ **WT**	**R*Cyp1b1***$^{-/-}$ **WT**
**R-*Cyp1b1***					
Cyp7a1	**845**	390	2.4**	nc	-5**
Nr0b2	**280**	60	4.5	2.2	+3.7*
Dmbt1	**1605**	24	80**	-25.4**	-14**
DBP	**14854**	3232	5.9**	-5.4**	-5*
Ppp1r3g	857	658	1.0	-7.3**	-3.4**
Acta2	**950**	350	3.2	-2.2**	- 3**
Scd1	**18785**	153	86.9**	**-12****	+2.2**
Sqle	**2365**	1434	2.4	-24**	nc
Gadd45g	**11356**	2175	6.2**	-6.6**	-1.8**
Rgs16	**3218**	1732	2.2**	-5.9**	2.0**

#Ratio determined by the Limma -Edge Algorithm.

Table S3. Body weights and expression for individual male and female mice divided by litter (A) and sex (B). A) *Acss2*, *Me1*, *Scd1*, *Fasn* and *Hamp.* Colors mark separate litters separated by M, F littermates. Litters are not complete due to random continuation of some pups for adult testing.

litter	BW	Litter	BW	*Acss*	*Acss*	*Me1*	*Me1*	*Scd1*	*Scd1*	*Fasn*	*Fasn*	*Hmp*	*Hmp*
Male		Female		M	F	M	F	M	F	M	F	M	F
	5L			<2.5		<2.5		<2.5		<2.5		<2.5	
BD													
5-5	10.4	12-4	9.8	2.4	1.8	2.3	2.3	-1.8	-3.1	2.5	1.0	-1.3	3.5
5-6	9.7	12-1	9.4	4.6	1.5	4.8	1.6	0.3	-2.6	3.6	0.55	-2.0	2.3
5-7	10.5	12-2	8.9	0.3	1.9	0.1	2.2	-0.9	-2.7	1.5	1.4	-1.3	2.5
37-2	9.5	12-3	7.8	5.8	1.5	4.9	2.8	1.6	-2.3	1.6	1.5	-1.8	3.9
37-3	8.6	37-1	8.6	3.0	3.8	3.9	3.9	2.8	0.3	2.9	2.0	3.4	0.0
		2-3	6.8		4.0		3.9		0.5		3.9		-0.2
		2-5	7.6		3.1		2.6		-1.4		1.4		-0.6
		3-3	7.7		3.2		2.7		-1.6		1.0		0.3
		3-4	8.4		3.9		2.7		-1.1		1.4		0.4
BD-*1b1*	3L												
51-2	6.7	11-1	9.7	nd	1.9	Nd	3.2	3.1	-1.9	5.1	1.7	-1.7	1.3
51-6	6.4	11-2	9.4	nd	2.4	Nd	4.3	3.1	-0.5	3.6	2.6	-1.1	0.9
73-2	7.8	11-3	9.1	4.0	2.9	3.9	4.4	3.0	-0.4	3.2	2.7	-0.3	0.3
		11-5	8.8		2.9		4.5		0.4		2.9		1.0
		11-6	9.1		3.0		3.2		-1.3		3.1		0.0
		51-1	7.4		2.7		3.7		0.4		1.6		-1.5
		51-3	6.8		4.4		4.0		2.1		3.2		-0.5
		51-4	6.9		3.9		3.9		0.5		3.2		-1.4
		73-1	7.7		5.5		4.3		3.2		3.9		4.3
		73-3	7.4		5.1		4.2		2.4		3.5		1.0
		73-4	8.4		3.8		3.7		0.4		1.8		-0.7
		73-6	7.9		5.6		3.7		1.0		3.7		1.4
LF12	6L												
32-2	7.8	32-1	8.5	2.0	2.4	2.4	1.9	-1.1	-2.0	1.8	0.6	3.4	2.3
71-1	6.0	32-3	7.0	5.1	3.1	5.2	3.1	3.5	-2.4	4.5	0.5	2.1	4.5
71-4	6.2	32-4	6.8	3.5	4.1	2.7	4.2	2.2	0.5	4.6	3.5	3.9	2.8
2-1	7.1	71-2	5.8	4.2	5.2	2.6	4.8	1.9	2.8	2.7	4.0	9.6	8.4
3-1	8.7	71-3	5.9	1.3	4.5	1.0	4.1	-2.6	0.9	-0.1	3.1	0.5	6.5
5;1	7-6			2.8		3.2		-1.3		1.9		-1.4	
5;2	6.8			4.3		3.0		1.7		3.2		5.7	
5;4	8.8			4.1		3.0		0.0		2.0		1.1	
6-1	7.9			1.6		1.7		-0.8		-0.8		1.2	
LF12 *1b1*	4L												
33-1	6.5	87-1	6.4	6.0	5.5	5.0	3.7	3.3	-0.9	3.3	4.7	5.1	2.3
33-6	6.7	87-2	6.3	5.7	4.7	5.2	4.3	3.4	1.3	3.4	3.3	8.0	9.2
769	6.9	87-4	6.4	4.5	4.2	2,8	4.3	2.9	1.5	2.6	3.2	8.4	8.2
769	6.5	33-2	6.3	5.5	4.9	3.6	4.2	3.8	3.1	4.1	2.9	10.1	10.8
769	6.8	33-3	6.2	5.1	4.9	3.3	4.7	3.3	2.1	3.7	3.6	7.6	9.3
773	7.2	33-4	6.5	5.8	4.9	4.7	3.8	2.8	0.8	4.0	2.9	6.1	8.9
773	6.7			5.5		4.5		4.0		3.8		6.9	
773	7.2			6.1		4.3		3.6		4.6		7.7	

87-3	6.0			5.1		4.6		3.3		3.3		8.4	
**LF-Fe**	**3L**												
**57-2**	6.5	80-1	9.0	5.1	0.5	3.7	0.4	3.0	-4.7	5.3	-0.2	1.2	2.2
**80-2**	9.4	80-3	8.7	2.0	0.7	1.1	1.1	-5.2	-4.2	-0.3	0.0	3.8	0.8
**80-4**	9.9	57-1	6.4	1.2	4.6	0.5	4.1	-4.6	-0.2	0.7	2.8	0.5	3.9
**80-6**	8.4	57-3	6.1	0.8	5.6	0.8	4.4	-4.5	3.7	-0.5	4.3	0.9	9.4
**91-2**	8.0	57-4	6.6	3.0	4.9	2.2	3.8	-2.9	2.1	1.3	2.7	1.1	1.3
**LF-Fe *1b1***	**3L**												
61-2	8.6	83-2	6.2	3.2	4.5	2.6	4.7	-0.8	2.5	1.9	3.7	-1.4	3.3
61-5	8.5	60-2	6.6	nd	3.8	nd	4.3	-0.4	0.9	3.3	2.3	-3.5	7.7
60-1	6.6	61-3	8.2	6.9	1.5	5.8	1.0	2.6	-3.0	5.2	0.2	-2.9	0.6
74-2	6.5	74-1	5.9	4.5	4.4	3.8	4.3	1.5	2.4	2.5	2.9	-2.5	2.6
74-4	5.9	74-3	6.1	5.0	5.1	4.9	2.9	2.2	0.4	0.7	2.9	-2.0	-0.2
83.5	6.4	74-5	6.5	4.5	4.3	2.5	2.5	1.1	-1.1	-1.4	2.1	3.1	-0.5
83.3	6.3	74-6	5.7	4.9	4.7	3.6	4.3	1.1	2.3	-0.6	2.9	9.5	4.7
83.1	6.0	74-7	5.7	5.3	3.9	3.6	3.6	1.6	0.9	-1.5	1.8	3.4	2.4

B) *Elovl6*, *Sqle*, *Hmgcr*, and *Hamp2*.

Litter	BW	litter	BW	*Elov6*	*Elov6*	*Sqle*	*Sqle*	*Hmgcr*	*Hmgcr*	*Hmp2*	*Hmp2*
**Male**		**Fem**		**M**	**F**	**M**	**F**	**M**	**F**	**M**	**F**
**BD**	**5L**			<4.0		<4.0		<4.0		<2.5	
**5-5**	10.4	12-4	9.8	3.4	3.7	5.3	4.5	3.2	3.6	-1.7	2.6
**5-6**	9.7	12-1	9.4	4.6	3.1	6.5	4.5	4.7	3.6	-1.8	1.5
**5-7**	10.5	12-2	8.9	3.8	4.1	2.6	4.7	0.9	3.7	-1.4	2.0
**37-2**	9.5	12-3	7.8	6.5	3.6	6.2	4.5	4.1	3.1	0.0	2.6
**37-3**	8.6	37-1	8.6	4.8	5.3	3.6	4.2	0.9	2.6	3.2	0 .8
		2-3	6.8		6.7		4.4		2.3		1.1
		2-5	7.6		3.6		4.0		1.4		1.0
**MF 37**		3-3	7.7		3.9		4.2		2.1		0.6
		3-4	8.4		3.9		6.0		3.1		1.2
**BD- *1b1***	3L										
**51-2**	6.7	11-1	9.7	7.5	4.0	3.9	4.8	3.0	3.8	-2.1	1.8
**51-6**	6.4	11-2	9.4	6.8	5.5	nd	4.4	nd	3.2	-1.5	0.6
**73-2**	7.8	11-3	9.1	6.0	5.4	4.3	4.4	2.0	3.4	0.6	0.2
		11-5	8.8		5.6		4.6		3.4		1.0
		11-6	9.1		5.5		4.4		3.2		-0.4
		51-1	7.4		6.1		4.7		2.9		-0.5
		51-3	6.8		7.8		6.5		4.1		0.4
		51-4	6.9		7.0		6.6		4.1		-0.2
		73-1	7.7		7.8		8.3		4.8		3.0
**M/F**	**51/73**	73-3	7.4		7.5		6.7		4.6		1.6
		73-4	8.4		5.9		4.8		3.0		0.8
		73-6	7.9		7.7		8.2		5.0		1.7
**LF12**	**6L**										
**32-2**	7.8	32-1	8.5	4.1	3.4	6.4	5.3	3.2	3.1	1.4	1.7

**71-1**	6.0	32-3	7.0	6.2	**3.5**	6.0	7.4	3.1	4.1	0.4	**2,2**
**71-4**	6.2	32-4	6.8	6.6	6.7	4.8	6.7	2.4	4.4	1.5	**1.2**
**2-1**	7.1	71-2	5.8	6.2	7.5	5.3	7.2	2.5	4.4	6.0	5.3
**3-1**	8.7	71-3	5.9	**2.2**	5.9	**2.9**	6.0	0.5	3.4	**0.8**	4.2
**5a-1**	7-6			4.3		4.1		1.3		-2.1	
**5a-2**	6.8			7.6		5.4		1.9		3.1	
**5a-4**	**8.8**			5.0		4.1		1.5		0.3	
**6-1**	7.9			**2.0**		**3.4**		**1.7**		3.3	
**M/F**	**32**<br>**71**										
**LF12**<br>***1b1***	4L			E		S		HM		HA	
**33-1**	6.5	87-1	6.4	7.8	6.8	7.2	8.3	4.3	5.0	2.7	1.7
**33-6**	6.7	87-2	6.3	6.4	6.6	5.7	4.5	3.7	3.3	4.0	6.0
**769**	6.9	87-4	6.4	6.5	6.7	7.1	4.2	3.0	3.3	3.8	4.8
**769**	6.5	33-2	6.3	7.7	6.4	9.5	5.6	4.1	3.9	4.0	6.5
**769**	6.8	33-3	6.2	7.2	7.2	9.2	6.1	3.8	3.7	4.2	4.2
**773**	7.2	33-4	6.5	7.2	6.1	9.9	6.0	4.5	4.4	3.3	5.0
**773**	6.7			6.8		9.5		3.8		4.2	
**773**	7.2			7.9		9.3		4.3		4.4	
**87-3**	6.0			6.5		5.5		3.5		5.4	
**M/F**<br>**33 87**											
**LF-Fe**	**3L**										
**57-2**	6.5	80-1	**9.0**	1.5	1.4	6.5	4.3	**4.0**	**nd**	**0.7**	1.3
**80-2**	**9.4**	80-3	8.7	2.2	1.6	**2.0**	**3.9**	**3.1**	**2.2**	3.1	0.8
**80-4**	**9.9**	57-1	6.4	1.4	5.6	**1.2**	6.5	**3.1**	4.4	0.6	4.1
**80-6**	8.4	57-3	6.1	2.1	7.7	**0.8**	7.7	**2.7**	5.4	2.5	6.1
**91-2**	8.0	57-4	6.6	1.2	6.3	5.8	6.3	**3.6**	4.4	**0.4**	**2.1**
**M/F**											
**57 80**											
**LF-Fe**<br>***1b1***	**3L**										
61-2	8.6	83-2	6.2	**3.1**	7.2	5.7	4.5	3.7	3.2	**-1.5**	**2.2**
61-5	8.5	60-2	6.6	4.4	5.3	nd	5.7	nd	3.4	**-2.1**	5.4
60-1	6.6	61-3	8.2	6.2	**1.9**	8.4	5.0	5.0	3.5	**-1.9**	**0.1**
74-2	6.5	74-1	5.9	5.4	6.2	5.9	4.7	2.5	3.3	**-2.0**	**1.7**
74-4	5.9	74-3	6.1	4.5	6.0	5.5	5.9	2.0	3.4	**-1.7**	**0.5**
83.5	6.4	74-5	6.5	3.0	4.4	5.8	5.6	2.6	3.7	2.6	**0.2**
83.3	6.3	74-6	5.7	3.0	7.1	6.3	4.6	3.1	2.8	5.7	5.3
83.1	6.0	74-7	5.7	2.7	5.5	5.9	4.5	4.5	2.7	3.2	**1.5**

Table S4. Effects of *Cyp1b1*$^{-/-}$ and Fe supplementation on Srebp activities and Hamp expression.

A) All litters averaged.

Litter	BW		*Acss2*	*Me1*	*Scd1*	*Fasn*	*Elov6*	*Sqle*	*Hmgcr*	*Hmp1*	*Hmp2*
**BD**	**8.7**		**3.0**	**2.9**	**-0.8**	**1.9**	**4.4**	**4.6**	**2.7**	**0.4**	**0.8**
**BD-*1b1***	7.9		3.7	3.9	1.0	3.0	6.4	5.6	3.9	0.2	0.5
d1b1	0.8		0.7	1.0	1.8	1.1	2.0	1.0	1.2	0.2	0.3
**LF12**	**7.5**		**2.8**	**2.6**	**-0.1**	**1.7**	**4.3**	**4.8**	**2.4**	**3.6**	**2.5**
**LF12/*1b1***	6.6		4.8	4.1	2.6	3.6	7.0	7.6	3.9	7.9	4.0
d1b1	0.9		2.0	1.5	2.7	1.9	2.7	2.8	1.5	4.3	1.5
**LF12 -Fe**	**7.8**		**3.1**	**2.3**	**-1.6**	**2.6**	**2.7**	**5.0**	**3.7**	**2.3**	**1.8**
**LF12-Fe-*1b1***	6.7		4.4	4.0	0.9	1.7	4.6	5.7	3.4	1.8	1.4
d1b1	1.1		1.3	1.7	2.5	0.9	1.9	0.7	Nc	-0.5	-0.4

B) Individual litters.

	BW		*Acss*	*Me*		*Fasn*	*Scd*	*Elv6*		*Sqle*	*Hmg*		*Ha1*	*Ha2*
**BD**														
**1**	10.2		2.4	2.4		**2.5**	-0.8	**3.9**		**4.8**	2.9		-1.5	-1.6
**2**	9.0		1.7	2.2		1.1	-2.7	**3.6**		**4.7**	3.5		3.1	2.2
**3**	8.9		4.2	4.2		**2.2**	**1.6**	**5.5**		3.7	2.5		0.5	1.3
**4**	8.1		**3.2**	**3.0**		1.2	-1.3	**3.9**		**5.1**	2.6		0.35	0.9
**5**	7.2		**3.6**	**3.2**		**2.6**	-0.5	**5.2**		4.2	1.8		-0.4	1.1
**BD-*1b1***														
**1**	9.2		**2.6**	3.9		**2.6**	-0.8	**5.2**		**4.5**	**3.4**		0.7	0.6
**2**	7.8		4.8	3.9		3.2	**2.0**	7.0		7.0	4.6		1.1	**1.7**
**3**	6.8		**3.7**	3.9		3.3	**1.9**	7.0		**5.9**	**3.7**		-1.2	-0.8
**LF12**														
**1**	8.7		1.3	1.0		-0.1	-2.6	2.2		2.9	0.5		0.5	0.8
**2**	7.9		1.8	1.7		-0.8	-0.8	2.0		3.4	1.7		1.2	3.3
**3**	7.7		**3.7**	**3.1**		**2.4**	**0.1**	**5.6**		4.5	1.6		1.8	0.5
**4**	7.5		**2.9**	**2.8**		1.6	-1.2	**4.4**		6.5	4.7		3.3	**1.6**
**5**	7.1		4.2	**2.6**		**2.7**	**1.9**	6.2		**5.3**	2.5		9.6	6.0
**6**	6.0		4.6	4.4		4.1	2.3	**5.5**		6.0	3.3		5.2	**2.8**
**LF12-*1b1***														
**1**	7.0		5.8	4.5		4.1	3.5	7.3		9.7	4.2		6.9	4.0
**2**	6.7		**3.2**	**3.2**		3.5	3.3	7.2		8.6	**3.6**		9.7	4.0
**3**	6.4		5.2	4.6		3.2	2.5	6.8		6.1	**4.0**		8.0	4.5
**4**	6.2		4.9	4.2		3.6	**1.3**	6.6		6.1	3.7		7.0	3.6
**LF12-Fe**														
**1**	9.1		1.0	0.8		3.5	-3.8	1.7		2.5	2.7		**1.6**	**1.7**
**2**	8.0		**3.0**	2.2		1.3	-2.9	1.2		**5.8**	**3.6**		1.1	0.4
**3**	6.4		5.2	4.0		**3.0**	**2.0**	**5.3**		6.8	4.7		4.2	**3.3**
**LF12-<br>*1b1*-Fe**														
1	7.7		3.8	**3.4**		**2.6**	-0.2	**4.2**		6.2	**3.9**		0.1	0.0
2	6.2		4.9	4.9		0.0	**1.6**	**4.0**		**5.6**	**3.4**		5.1	**3.4**
3	6.1		4.6	3.8		**2.6**	**1.4**	**5.6**		**5.3**	2.9		0.2	0.8

Color Ranges:								
	Acss2/Me1	1.0- 2.6	**2.7-3.9**	4.0 -5.8	*Fasn*	-0.1 -1.6	**1.7-3.0**	3.1-5.66
	Scd1	-3.8 - -0.1	**0.0-2.0.**	2.1-5.2	*Elovl6*	1.2- 3.3	**3.4-5.6**	5.
	Hmgcr	0.5 - 2.9	**3.0 - 4.1**	4.2 -8.9	*Hamp1/Hamp2*	-1.6 - 1.2	**1.3-2.9,** >	2.9-

Table S5. LF12 defined diets.

Formula	LF12	LF12 + Fe	LF12 + FSO
	g/Kg	g/Kg	g/Kg
Casein	199.0	190.0	190.0
L-Cystine	3.0	3.0	3.0
Corn Starch	393.2	392.9	386.18
Sucrose	150.0	150.0	150.0
Maltodextrin	130.0	130.0	130.0
Cottonseed Oil	50.0	50.0	50.0
Flaxseed Oil	0.0	0.0	**7.0**
Cellulose	40.0	40.0	40.0
Mineral Mix, AIN-93G-MX	35.0	35.0	35.0
Vitamin Mix, w/o choline, A, D, E	5.0	5.0	5.0
Ferrous Sulfate, heptahydrate	0.0	**0.3**	0.0
Choline Dihydrogen Citrate	3.5	3.5	3.5
Vitamin E, DL-alpha tocopheryl acetate (1000 IU/g)	0.121	0.121	0.121
Vitamin D3, cholecalciferol (4000 IU/g in sucrose)	0.0055	0.0055	0.0055
Vitamin A Palmitate (500,000 IU/g)	0.048	0.048	0.048
